# Wide spectrum and high frequency of genomic structural variation, including transposable elements, in large double-stranded DNA viruses

**DOI:** 10.1093/ve/vez060

**Published:** 2020-01-27

**Authors:** Vincent Loiseau, Elisabeth A Herniou, Yannis Moreau, Nicolas Lévêque, Carine Meignin, Laurent Daeffler, Brian Federici, Richard Cordaux, Clément Gilbert

**Affiliations:** 1 Laboratoire Evolution, Génomes, Comportement, Écologie, Unité Mixte de Recherche 9191 Centre National de la Recherche Scientifique et Unité Mixte de Recherche 247 Institut de Recherche pour le Développement, Université Paris-Saclay, Gif-sur-Yvette 91198, France; 2 Institut de Recherche sur la Biologie de l'Insecte, UMR 7261 CNRS - Université de Tours, 37200 Tours, France; 3 Laboratoire de Virologie et Mycobactériologie, CHU de Poitiers, 86000 Poitiers, France; 4 Laboratoire Inflammation, Tissus Epithéliaux et Cytokines, EA 4331, Université de Poitiers, 86000 Poitiers, France; 5 Modèles Insectes d’Immunité Innée (M3i), Université de Strasbourg, IBMC CNRS-UPR9022, Strasbourg F-67000, France; 6 Department of Entomology and Institute for Integrative Genome Biology, University of California, Riverside, CA 92521, USA; 7 Laboratoire Ecologie et Biologie des Interactions, Equipe Ecologie Evolution Symbiose, Unité Mixte de Recherche 7267 Centre National de la Recherche Scientifique, Université de Poitiers, 86000 Poitiers, France

**Keywords:** large double-stranded DNA viruses, genomic structural variation, transposable elements, herpesvirus, iridovirus, baculovirus

## Abstract

Our knowledge of the diversity and frequency of genomic structural variation segregating in populations of large double-stranded (ds) DNA viruses is limited. Here, we sequenced the genome of a baculovirus (*Autographa californica* multiple nucleopolyhedrovirus [AcMNPV]) purified from beet armyworm (*Spodoptera exigua*) larvae at depths >195,000× using both short- (Illumina) and long-read (PacBio) technologies. Using a pipeline relying on hierarchical clustering of structural variants (SVs) detected in individual short- and long-reads by six variant callers, we identified a total of 1,141 SVs in AcMNPV, including 464 deletions, 443 inversions, 160 duplications, and 74 insertions. These variants are considered robust and unlikely to result from technical artifacts because they were independently detected in at least three long reads as well as at least three short reads. SVs are distributed along the entire AcMNPV genome and may involve large genomic regions (30,496 bp on average). We show that no less than 39.9 per cent of genomes carry at least one SV in AcMNPV populations, that the vast majority of SVs (75%) segregate at very low frequency (<0.01%) and that very few SVs persist after ten replication cycles, consistent with a negative impact of most SVs on AcMNPV fitness. Using short-read sequencing datasets, we then show that populations of two iridoviruses and one herpesvirus are also full of SVs, as they contain between 426 and 1,102 SVs carried by 52.4–80.1 per cent of genomes. Finally, AcMNPV long reads allowed us to identify 1,757 transposable elements (TEs) insertions, 895 of which are truncated and occur at one extremity of the reads. This further supports the role of baculoviruses as possible vectors of horizontal transfer of TEs. Altogether, we found that SVs, which evolve mostly under rapid dynamics of gain and loss in viral populations, represent an important feature in the biology of large dsDNA viruses.

## 1. Introduction

Estimating the evolutionary potential of viral populations is key to our understanding of how and how fast viruses may evolve in response to new environmental constraints. Such potential is directly linked to the genetic diversity of viral populations, which has been well characterized in only a handful of viruses. RNA viruses display high mutation rates, large population sizes, and fast replication dynamics, which all together generate clouds of genetically linked single nucleotide variants that functionally cooperate and collectively contribute to the fitness of the viral population (Lauring and Andino 2010; Acevedo, Brodsky, and Andino 2014). Such extremely high levels of polymorphism allow RNA viruses to rapidly adapt to the various host and cellular environments they may be exposed to (Lauring, Frydman, and Andino 2013; Sanjuán and Domingo-Calap 2016). It also makes the outcome of infection difficult to predict, and thus poses major challenges to the prevention and treatment of viral diseases.

In contrast to most RNA viruses, which do not encode error-correcting polymerases, large double-stranded DNA (dsDNA) viruses use high-fidelity, proofreading polymerases (Duffy, Shackelton, and Holmes 2008; Sanjuán and Domingo-Calap 2016). As a consequence, the mutation rate of large dsDNA viruses is two to four orders of magnitude lower than that of RNA viruses. Despite these lower mutation rates, populations of large dsDNA viruses exhibit very high nucleotide diversity. For example, high-throughput sequencing approaches have revealed several thousands of single nucleotide polymorphisms (SNPs) segregating in populations of the human cytomegalovirus (HCMV) (Renzette et al. 2013, 2015, 2017), the *Autographa californica* multiple nucleopolyhedrovirus (AcMNPV) (Chateigner et al. 2015), and human herpesvirus 2 (Akhtar et al. 2019). Although the majority of these SNPs are at low frequency and likely neutral, a fraction was shown to be under positive selection and involved in rapid adaptation during intra-host evolution (Renzette et al. 2013).

In addition to SNPs, another source of genetic diversity found in viral populations is structural variation, which may be defined as deletions, insertions, inversions, duplications and translocations (Alkan, Coe, and Eichler 2011). Some forms of structural variants (SVs), leading to defective viral genomes (DVGs), have been the subject of extensive experimentation because of the negative impact they have on viral replication (Marriott and Dimmock 2010). DVGs were first discovered in populations of the Influenza A virus and have since been extensively studied in RNA viruses (Manzoni and López 2018). Their presence in RNA viral populations is pivotal to the intra-host dynamics of viral infections, to the point that abnormal depletion in DVG can lead to severe disease outcomes (Vasilijevic et al. 2017). Such DVGs, provided they contain all the signals necessary for packaging, can outcompete complete viral genomes and rapidly cause important drops in overall virus titers (Li et al. 2011). RNA virus DVGs are also known to play a role in the induction of the interferon-mediated antiviral response (Lopez 2014) as well as in the Dicer-dependent viral DNA-mediated antiviral RNAi response in insects (Poirier et al. 2018).

Historically, DVGs have been less studied in large dsDNA viruses. With the development of protein expression vectors, most experiments focused on baculoviruses (De Gooijer et al. 1992). Experimental assays coupled to population genetics modeling characterized interactions between complete and DVGs (Bull, Godfray, and O'Reilly 2003; Zwart, Tromas, and Elena 2013) to assess what proportion of DVGs may be optimal to limit the persistence of complete viruses used as biopesticides (Kool et al. 1991; Godfray, Reilly, and Briggs 1997). These approaches mostly revealed a negative impact of DVGs on virus replication and production. Yet, in natural viral populations beneficial interactions may exist between defective and complete viral genomes, as mixtures are more pathogenic than clonal wild type populations (Simón et al. 2006). Besides baculoviruses, a high proportion of non-canonical viral genomes have also been detected in populations of human herpesviruses using molecular combing or Sanger sequencing (Mahiet et al. 2012).

Despite the impact of SVs on viral population dynamics and infection outcome, our knowledge on their full spectrum and frequency remains limited. Next generation sequencing (NGS) offers potent tools to probe the extent of SV diversity in large viral populations (Acevedo and Andino 2014). However, most studies of viral SVs using NGS have so far focused on major variants through assembling and comparing consensus genomes from different viral strains (Szpara et al. 2014; Karamitros et al. 2016, 2018). Surveys of intra-host viral SVs, as detected in individual sequencing reads, remain scarce, and often limited to specific, targeted rearrangements (Elde et al. 2012; Sasani et al. 2018). One of the most comprehensive NGS-based analysis of SVs diversity has been conducted on the flock house virus (FHV; *Alphanodaviridae*) after replication in *Drosophila melanogaster* S2 cells (Routh et al. 2015; Jaworski and Routh 2017). These studies led to the characterization of hundreds of different recombination events along the FHV RNA1 and FHV RNA2 genome segments and unveiled the precise dynamics and mechanisms underlying the emergence of DVGs during serial passaging of the virus in cell culture over a 1-month period.

One limitation of NGS to study SVs is the well-known propensity of both long- and short-read sequencing technologies, to generate artificial chimeras, which are difficult to distinguish from biological recombination events, during library construction (Tsai et al. 2014; Griffith et al. 2018; Peccoud et al. 2018). In addition, the quantity of viral particles that are directly recovered from natural hosts is often relatively small, which makes it difficult to purify enough viral DNA to prepare sequencing libraries. All NGS studies of SVs in viral populations have thus so far been done using viruses passaged in cell lines. Here, we sought to estimate the diversity of SVs that segregate in large dsDNA virus populations following natural host infections. First, we sequenced a large population of AcMNPV genomes purified from *Spodoptera exigua* larvae using both short-read Illumina and long-read PacBio sequencing technologies in parallel. Using a novel pipeline involving hierarchical clustering of SVs detected by six variant callers, we counted SVs present in both sequencing datasets. As PacBio and Illumina technologies are subject to different biases, we reasoned that SVs retrieved from both datasets are unlikely to derive from technical artifacts and can be considered robust. Based on the results obtained for AcMNPV, we then estimated SVs in populations of two other invertebrate large dsDNA viruses, the invertebrate iridescent virus 31 (IIV31) and 6 (IIV6) extracted from adults of the pillbug *Armadillidium vulgare* and from larvae of the moth *Sesamia nonagrioides*, respectively, and in a population of the HCMV purified from MRC5 cells.

## 2. Materials and methods

### 2.1 Infection of *S. exigua* larvae with AcMNPV

The AcMNPV-WP10 isolate (Chateigner et al. 2015) was used to infect 150 fourth instar larvae of the beet armyworm (*S.* *exigua*) using the diet plug method (Sparks, Li, and Bonning 2008). Each moth larva was fed ∼100,000 occlusion bodies (OBs) per 5 mm^3^ diet plug. Upon host death, which occurred 2–5 days post-infection, OBs were first filtered through cheesecloth, purified twice by centrifugation (10 min at 7,000 rpm) with 0.1 per cent sodium dodecyl sulfate, then distilled water, and finally resuspended in water. Approximately 1.5 × 10^10^ OBs were treated as described in Gilbert *et al.* (2014) to provide about 50 µg of high-quality dsDNA (about 5.82 × 10^11^ genomes assuming 100 genomes per OB; Ackermann and Smirnoff, 1983; Slack and Arif, 2006; Rohrmann, 2014). Briefly, OBs were purified by a percoll gradient at pH 7.5, sucrose 0.25 M (9 V of percoll/sucrose solution were added to 1 V of virus solution) with a centrifugation step (30 min at 15,000 g, 4°C). OBs were dissolved using Na_2_CO_3_ to release nucleocapsids (O’Reilly, Miller, and Luckow 1992). Viral DNA was then extracted using the QIAamp DNA Mini kit (Qiagen).

### 2.2 Infection of *A. vulgare* with IIV31

A solution containing IIV31 viral particles was obtained through grinding a piece of cuticle from a naturally infected *A.* *vulgare* individual collected on the campus of the University of California Riverside. One *A. vulgare* individual was pricked with a thin needle soaked in the viral solution. Fourteen days after the infection, the pillbug became bluish and died about 4 weeks after infection, as described in Lupetti et al. (2013). Upon death, the pillbug was crushed with a pestle and put in a 1.5 ml Eppendorf tube in a Tris solution. An ultra-centrifugation step on sucrose cushion was then performed at 35,000 g for 90 min at 4°C. The pellet was resuspended in 100 µl of Tris solution. Viral DNA was then extracted using the QIAamp DNA Mini kit (Qiagen).

### 2.3 Infection of *S. nonagrioides* larvae with IIV6

Ten fourth instar larvae of the Mediterranean corn borer *S. nonagrioides* were infected with the IIV6 viral strain originally described in Fukaya and Nasu (1966 ). Larvae were pricked using a thin needle soaked in the viral solution. Fourteen days later, the larvae presented a purple iridescence and they finally died about four weeks after infection. Upon host death, viral particles were filtered through cheesecloth and two centrifugation steps were performed to eliminate most of host cells and tissues. Then, an ultra-centrifugation step was performed as described above for IIV31. Viral DNA was then extracted using the QIAamp DNA Mini kit (Qiagen).

### 2.4 Infection of MRC5 cells with HCMV

MRC5 human fibroblasts were cultured in Dulbecco’s modified Eagle medium (Invitrogen) supplemented with 10 per cent fetal bovine serum, 4.5 g/l glucose, and 1 per cent penicillin-streptomycin (Pen-Strep; Life Technologies) at 37°C in a 5 per cent (vol/vol) CO_2_ atmosphere. Before HCMV infection, MRC5 cells were grown to confluence, resulting in ∼3.0 × 10^4^ cells per cm^2^. Once confluent, the medium was removed, and serum-free medium was added. Cells were maintained in serum-free medium for 24 h before infection at which point, they were infected at a multiplicity of infection of 10 pfu/cell with a clinical strain of HCMV isolated from a patient in 2015. After a 2 h adsorption period, the inoculum was aspirated, and fresh serum-free medium was added. Cells were harvested 8 days after infection through trypsinization followed by washing in Earle’s balanced salt solution and centrifugation at 1,100 g. Pelleted cells were then transferred into a 15 ml Falcon tube and cell lysis was performed by several steps of freeze/thaw cycles in dry ice and water bath at 37°C. The solution was centrifuged at 5,000 g for 30 min at 4°C and the supernatant containing viral particles was collected. Purification of viral particles and viral DNA extraction was performed as described above for iridoviruses.

### 2.5 Sequencing

For each virus, an aliquot containing 2 µg of DNA was used to construct a paired-end library (insert size 260 bp), which was sequenced on a Illumina HiSeq™ 2500 machine (Illumina, San Diego, CA, USA), generating 298, 298, 582, and 308 million 151-bp paired reads for AcMNPV, HCMV, IIV6, and IIV31, respectively. For PacBio sequencing, about 15 µg of AcMNPV DNA was used to construct one library. This library was sequenced at the McGill University and Genome Quebec Innovation Center on eight SMRT cells using the PacBio Sequel instrument, which generated 3 million reads (31 Gb).

### 2.6 Assembly and annotation of the consensus viral genomes

A consensus viral genome was assembled for all four viruses sequenced in this study. For AcMNPV, the viral assembly was based on the long reads that altogether reached a 203,467× depth of the AcMNPV genome. All PacBio reads longer than 30 kb (68,173 out of 3,012,899 reads, corresponding to 21× coverage depth on the viral genome) were assembled with Canu v1.5 (Koren et al. 2017; main options: -d AsmCanu-auto genomeSize = 134k -pacbio-raw). The raw assembly was then polished with Pilon v1.22 (Walker et al. 2014; default options) using the short reads that altogether reached a 196,093× depth of the AcMNPV genome. The polished assembly was then circularized with ToAmos v3.1.0 (Treangen et al. 2011) and minimus2 (Sommer et al. 2007) with default options. The assembly was then annotated based on the AcMNPV-E2 strain genome (accession number KM667940.1) with the General Annotation Transfer Utility program (Tcherepanov, Ehlers, and Upton 2006). The HCMV, IIV6, and IIV31 genomes were assembled as follows. For each virus, read subsamples corresponding to 500 and 1,500× depth coverage were assembled with tadpole (https://jgi.doe.gov/data-and-tools/bbtools/bb-tools-user-guide/tadpole-guide/, version of December 2018, options used: ‘k = 17’; ‘k = 31’; ‘k = 60’; ‘k = 90’ with ‘mincov = 100’). The different assemblies obtained with 17, 31, 60, and 90 mers with 500 and 1,500× depth coverage were then assembled with Geneious version 11.0.2 (https://www.geneious.com, options: de novo assembly, Geneious assembler, high sensitivity). The final assemblies were annotated based on the available HCMV (NC_006273.2), IIV6 (NC_003038.1), or IIV31 (NC_024451.1) genomes with the General Annotation Transfer Utility program (Tcherepanov, Ehlers, and Upton 2006).

### 2.7 SV detection

Illumina reads were aligned on the viral genomes assembled in this study using BWA (Li and Durbin 2009, options: -R ‘@RG\tID: id\tSM: sample\tLB: lib’) and blastn (options: -outfmt 6 -max_target_seqs 2 -max_hsps 2). SVs were called with four different pipelines: Pindel (Ye et al. 2009), Lumpy (Layer et al. 2014), Fermikit (Li, 2015) and a custom Python script. Pindel and Lumpy were run on bam files produced by BWA (options: -R ‘@RG\tID: id\tSM: sample\tLB: lib’). Fermikit is an SV caller based on local read assembly, which uses raw reads as input. The custom python script is derived from that used in Chateigner et al. (2015) to find large deletions using short-read pairs. The script runs on tabular blastn output files and identifies deletions by comparing the observed distance separating both reads of a pair with the distance expected according to the mean library insert size. To account for experimental variation in insert size not due to structural variation, we only considered inter-read distances longer than 700 bp as reflecting true SVs (Supplementary Fig. S1). Deletions of smaller genome fragments cannot be confidently identified with this script. All read pairs involved in a deletion event of approximately the same start position, end position (maximum length between start positions or end positions was 7 bp) and length were clustered in SV events each characterized by an average start and end position, as well as an average length and a number of read pairs supporting the deletion event. As we had no expectation regarding the final number of clusters, we followed previous studies (Mönchgesang et al. 2016; Parikh et al. 2016; Zhang et al. 2018) and used a hierarchical clustering method rather than the K-means method, which is based on a known number of clusters. Briefly, Euclidean distances of start and end positions were computed between all SVs to generate a distance matrix. Then the linkage step between SVs was performed according to the Ward method (Ward 1963). The threshold value was automatically defined using the inconsistency method (Jain and Dubes 1988). Clustering was performed with the ‘scipy.cluster.hierarchy’ Python package (https://docs.scipy.org/doc/scipy/reference/cluster.hierarchy.html).

PacBio reads were aligned on the AcMNPV genome using BWA and SVs were called on the bam file with sniffles (Sedlazeck et al. 2018). SVs were also called with PbHoney (English, Salerno, and Reid 2014), which takes raw long reads as input. Both SV callers were run using default parameters. All SV caller output files were treated as Variant Call Format files in downstream analyses.

### 2.8 SV analyses

**Figure 1.**
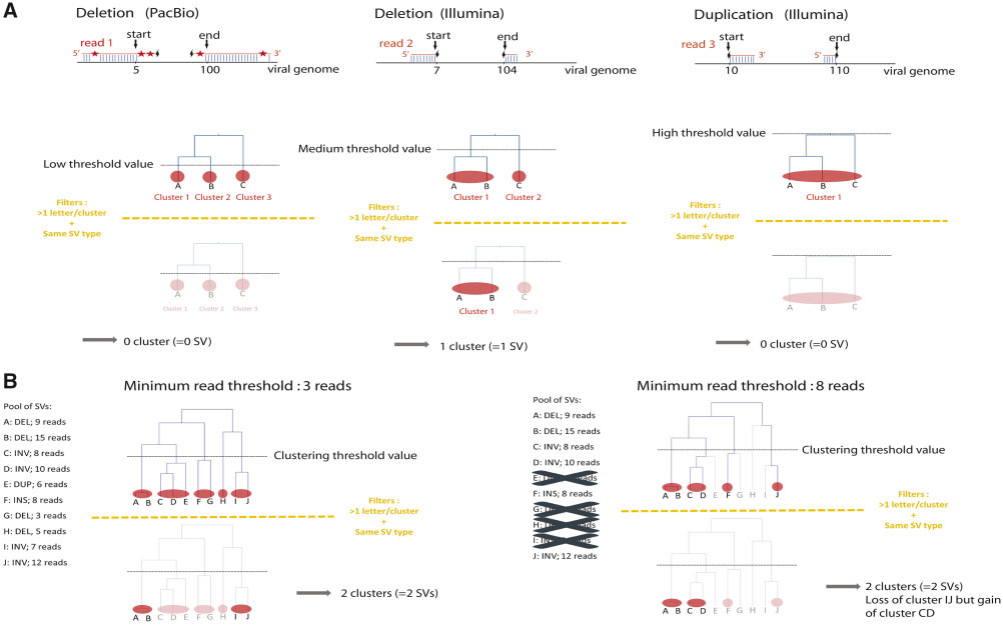
Illustration of two important steps of the hierarchical clustering of SVs. (A) Influence of the clustering threshold value. The top panel illustrates three reads (one long PacBio read and two short Illumina reads) mapped onto overlapping regions of the viral genome. Red asterisks correspond to sequencing errors that prevent accurate mapping of long reads. ‘start’ and ‘end’ correspond to start and end coordinates of the SV detected by SV callers (a deletion in the case of Reads 1 and 2 and a duplication for Read 3. The bottom panel shows how using multiple clustering thresholds prevents discarding well-supported SVs. With a low threshold, all clusters contain a single SV because none of the SVs have the exact same coordinates. Because a downstream filter of our pipeline requires that SVs must be detected either by both long and short reads (in the case of the AcMNPV population sequenced using both Illumina and PacBio technologies) or by two programs (in the case of the three other large dsDNA viruses sequenced only with Illumina) to be retained, none of the SVs are retained with this low clustering threshold. With a high clustering threshold, all SVs (two deletions and one duplication) end up in the same cluster because they are defined by coordinates that are close to each other. Because a downstream filter of our pipeline requires that all SVs within a cluster must be of the same nature for a cluster to be retained, the cluster is here not considered further. With a medium threshold, the deletions detected by Reads 1 and 2 are lumped into the same cluster because their coordinates are close enough and the duplication detected by Read 3 forms another cluster because its coordinates are too far from those of the deletion. After running the downstream filters of our pipeline, Cluster 2 is retained and one deletion is counted because it has been detected independently by long and short reads. The cluster containing the duplication is not considered further because it contains only one SV detected by short reads only. Note that although SVs supported by only one read are represented here for the sake of simplicity but our approach only retained SVs supported by a minimum of three reads. (B) Influence of the minimum number of reads supporting a SV. On the left panel, using three reads as the minimum number of reads required to retain SVs, ten SVs of different nature and/or supported by different numbers of reads have been detected by SV callers. Under a given clustering threshold value, these ten SVs form five clusters, only two of which are retained (A–B and I–J) by downstream filters because they contain several SVs which are all of the same nature. On the right panel, only six of the ten SVs detected on the left panel are detected by SV callers using eight reads as the minimum number of reads required to retain SVs. With the same given clustering threshold value as in the left panel, SVs form four clusters, two of which (A, B and C, D) are retained by downstream filters because they contain several SVs which are all of the same nature. Using multiple minimum numbers of reads supporting SVs ensure that well-supported SVs (here the inversion in C and D) are not eliminated by downstream filters.

To remove redundancy in SVs (a given SV may be detected by more than one SV caller), all SVs supported by three reads or more were clustered using a hierarchical clustering approach implemented in the ‘fastcluster’ R package (Müllner 2013), the R version of the Python package used for the custom SV Python SV caller. To take into account the relative imprecision in the coordinates of some clusters, we did not define clusters based on the inconsistency method, instead we performed multiple rounds of clustering (fourteen rounds, see below), each time using a different threshold value (Fig. 1A). The use of different thresholds allowed us to take into account the fact that high threshold values can induce erroneous clustering of different SV events which coordinates are very close to each other. Importantly however, clusters containing different types of SVs (e.g. a deletion and an inversion which have the same coordinates) are removed from the analyses. On the other hand, small threshold values can miss clustering of identical SVs detected by different programs due to slight differences in coordinate precision between programs, as more particularly noted in the case of the AcMNPV long-read dataset. The error-prone long reads can be mapped approximately due to artefactual SNPs and insertions/deletions (indels) present in the reads. Such approximations can lead to different start and end coordinates for a same SV between the different sequencing technologies and the different alignment programs. Due to these slight differences in coordinates for the same SV, a low threshold value will not cluster these different coordinates sets into one cluster but will give many clusters each with one pair of coordinates. With only one threshold value, downstream filters would often erroneously remove some clusters (i.e. some SVs) because all clusters supported by only one SV caller are not considered robust and discarded in our approach. For example, if a deletion was detected with a long-read SV caller at coordinates 5–50 and with a short-read SV caller at coordinates 6–54, a too low threshold value would not cluster both coordinate sets in one cluster (one SV) supported by the two SV callers but it would cluster them in two different clusters each supported by one SV caller. Then, a filter in downstream analyses would remove all clusters not supported by at least one long-read and one short-read SV callers. Thus, the two SVs detected would be erroneously removed whereas they in fact correspond to the same biological SV but with slightly different mapping coordinates. With a higher threshold, these two SVs detected would be clustered together and kept as one SV. That is why we used different threshold values.

To avoid redundancy in SV detection due to the use of many threshold values, duplicated SVs were removed. For each dataset, we performed a total of fourteen different clustering steps each with a different threshold value (5; 10; 30; 50; 100; 200; 300; 400; 500; 600; 700; 800; 900; and 1,000). To further improve the delineation between different SVs that may involve close genome breakpoint coordinates; we included read coverage in our analysis, reasoning that different SVs may often be supported by a different number of reads (Fig. 1B). Thus we repeated the above-described round of clustering (involving all different fourteen clustering threshold values) twenty-two times using twenty-two different thresholds for the minimal number of reads supporting each SV (4; 5; 6; 7; 8; 9; 10; 20; 30; 40; 50; 100; 200; 300; 400; 500; 600; 700; 800; 900; 1,000; and 1,500). The number of merged SVs differed depending on these different threshold values. Some SVs could be removed by downstream filters when too many discordant SVs were merged (more likely when the minimum read number threshold was low) whereas the same SVs had a lower chance to be removed by downstream filters when higher numbers of reads were used, inducing a less aggressive clustering. All SVs obtained through these clustering steps were retrieved and a final list of SVs was established after removing redundancy that is, all identical SVs found under different thresholds were counted only once.

For the AcMNPV virus, the clustering procedure was performed jointly on the Illumina and PacBio datasets. Also, to avoid false SV discovery due to a detection error caused by the circular nature of the AcMNPV genome, additional filters were added for this virus. Some long reads involved in the SVs were retrieved and aligned on the AcMNPV genome with Geneious version 11.0.2 (https://www.geneious.com). Some of them corresponded to the start and end coordinates of the AcMNPV consensus genome, thus did not capture an SV event and overestimated the number of reads supporting an SV. Empirically, we found that false SVs were mainly supported by a few number of reads (ten to twenty reads). SVs with a length >67 kb (half the size of the AcMNPV genome) and supported by <20 long reads or by <3 SV callers were discarded from the analysis. After obtaining a final list of SVs for each virus, average start position, end position and length were calculated for each SV. Finally, viral genes corresponding to the average start and end SV positions were identified based on the viral genome general feature format file.

### 2.9 SV frequencies in viral populations

Our calculation of the SV frequency was based on the approach commonly used to calculate SNP frequency that is, SNP coverage/(SNP coverage + reference coverage) at the SNP position. Thus, we calculated SVs frequency as follows: SV coverage/(SV coverage + reference coverage) at the SV position, using a per-base coverage file of all alignments obtained with bedtools genomecov (Quinlan and Hall 2010; option: -d) for the four SV callers relying on the use of a bam mapping file.

### 2.10 Simulation of AcMNPV short reads

We simulated a mock dataset of short reads from the AcMNPV genome with 200,000× depth, equivalent to our real dataset. The mock reads were generated with the Grinder program (Angly et al. 2012) with point mutations and chimeras, to mimic a real Illumina dataset (options: ‘-coverage_fold 200,000 -read_dist 150 uniform 0 -insert_dist 230 normal 50 -mate_orientation FR -chimera_perc 5 -chimera_dist 1 -chimera_kmer 0 -mutation_dist uniform 0.3 -mutation_ratio 99.7 0.3’). The simulation yielded 89,322,000 150-bp reads, on which we ran our SV detection pipeline.

### 2.11 Characterization of SVs in twenty-one AcMNPV datasets

A published experimental evolution dataset of AcMNPV, whereby a population of this virus purified after several rounds of infection on the cabbage looper moth (*Trichoplusia ni*) was Illumina-sequenced at 187,536× average depth and was independently passaged in ten lines of *T. ni* larvae and ten lines of *S. exigua* larvae, each line consisting of ten successive infection cycles (Gilbert et al. 2016). AcMNPV OB’s recovered from the last infection cycle of each of twenty evolved AcMNPV populations were sequenced at between 9,211 and 33,783× average depth for the ten *T. ni* lines (total depth = 145,386×) and between 3,497 and 35,434× average depth for the ten *S. exigua* lines (total depth = 163,610×). To detect SVs in each of the twenty-one AcMNPV Illumina datasets, we applied the method described above for the AcMNPV Illumina dataset, involving hierarchical clustering of the outputs of four SV callers. As long reads were not available for any of these twenty-one datasets, we restricted our analysis of SV frequency to the 4.98 per cent most robust SVs detected in each dataset by selecting SVs supported by two variants callers (in line with the 4.98 per cent SVs jointly detected in both short and long reads among all SVs in the first analysis above). When the number of SVs supported by two variant callers was <4.98 per cent of all SVs, we selected all SVs detected by two variant callers plus another set corresponding to the most frequent SVs to reach 4.98 per cent.

### 2.12 Transposable element insertions

Our search for host sequences integrated into viral genomes involved aligning viral reads on various databases of publicly available sequences from the very host species used in this study or from species related to these hosts. For *S. nonagrioides*, our database included all nuclear and mitochondrial genomic and transcriptomic data of all lepidopterans available in GenBank as of 20 January 2018 (Benson et al. 2005) and the databases of all beet armyworm and cabbage looper contigs used in Gilbert *et al.* (2016). To increase our chances to detect host transposable element (TE) insertions, we used all TE sequences available in Repbase as of 15 October 2017 (Bao, Kojima, and Kohany 2015) and those identified with RepeatModeler (http://www.repeatmasker.org) in 196 insect genomes in Peccoud *et al.* (2017). For human, we used the GRCh38.p12 version of the human genome (GenBank assembly accession: GCA_000001405.27). For the pillbug, we used the *A. vulgare* genome (Chebbi et al. 2019). For the different host/virus systems studied, we also retrieved non-viral reads and assembled them with the SPAdes assembler (Bankevich et al. 2012). Then we aligned the resulting contigs on the GenBank nr database. We also aligned these contigs against themselves to search for terminal inverted repeats or long terminal repeats that are specific sequences found at the end of full-length TE sequences (Craig, 2002). Contigs corresponding to full-length TE sequences were added to previous databases to refine the search for TEs integrated in viral genomes.

Junctions between viral and host sequences were searched in Illumina short reads following Gilbert et al. (2016). Briefly, the raw Illumina reads were trimmed to remove adapters. Then they were aligned separately to host genomic and transcriptomic databases and to the viral genome using blastn (option ‘megablast’). Only reads aligning over at least 16 bp on the viral genome only and over at least 16 bp on a host sequence only were retained. Reads had to align on at least 130 bp (out of a total length of 151 bp) of their length. The overlap between alignment on the virus and on the host sequences was set to involve at most 20 bp and at least −5 bp (see Supplementary Fig. S6 in Gilbert et al. 2016).

Host sequences integrated into AcMNPV genomes were further searched by mapping long PacBio reads on the host databases with BLASR (Chaisson and Tesler 2012). BLASR tabular outputs obtained for each host database were merged, overlapping hits were identified and among them only the best-score hit was retained. Regions not mapping on host sequences were aligned to the viral genome with BLASR program to validate the host/virus chimeric nature of the reads.

The observed proportion of TE sequences at read ends was calculated by counting the number of TE sequences that were at a read end among all the TE sequences. The expected proportion of TE sequences at read ends was calculated by dividing the total TE sequences length by the total read length. A binomial test was performed to compare the observed and expected proportions of TE sequences at read ends. Statistical analyses were performed in R version 3.4.4  (R Core Team 2018).

## 3. Results

### 3.1 AcMNPV consensus genome

Our hybrid assembly of the AcMNPV genome yielded a 133,981-bp consensus genome which is 99.92 per cent identical to and 15 pb longer than the AcMNPV-E2 strain (Maghodia, Jarvis, and Geisler 2014). Both genomes diverge by sixty-three SNPs and sixteen short (<10 bp each) indels. All sixteen indels are supported by >80 per cent of Illumina reads covering these variants. These indels affect eight genes and disrupt the open reading frame in five of them (Ac-bro, Ac-odv-e18, Ac-gp64, AcOrf-91, and Ac-lef4). The sixty-three SNPs involve fourteen genes (AcOrf-34, AcOrf-18, Ac-IE-1, Ac-49K, Ac-IE-0, Ac-ME53, Ac-chitinase, AcOrf-114, Ac-helicase, AcOrf-74, Ac-lef3, A-lef8, Ac-pcna, and Ac-odv-e66). Only two of these sixty-three SNPs are fixed in the population, whereas the remaining sixty-one coexist at high frequencies (65.0–98.5%) with the alternative variant of the AcMNPV-E2 genome.

### 3.2 Nature, number and frequency of SVs in the AcMNPV population

**Figure 2.**
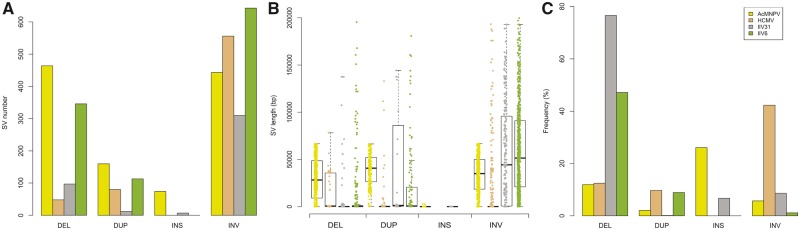
Number, size, and frequency of SVs in the four viral populations. (A) Number of detected SVs by SV type for the four viruses. No insertions were detected in the HCMV and IIV6 viral population. Insertions were only detected in long-read AcMNPV and in the IIV31 short reads. (B) Boxplots representing the size of detected SVs by SV type for the four viral populations. (C) Frequency of viral genomes carrying SVs shown by SV type for the four viral populations. The frequency was computed considering SV number per viral genome follows a Poisson distribution. DEL, deletion; DUP, duplication; INS, insertion; INV, inversion.

**Figure 3.**
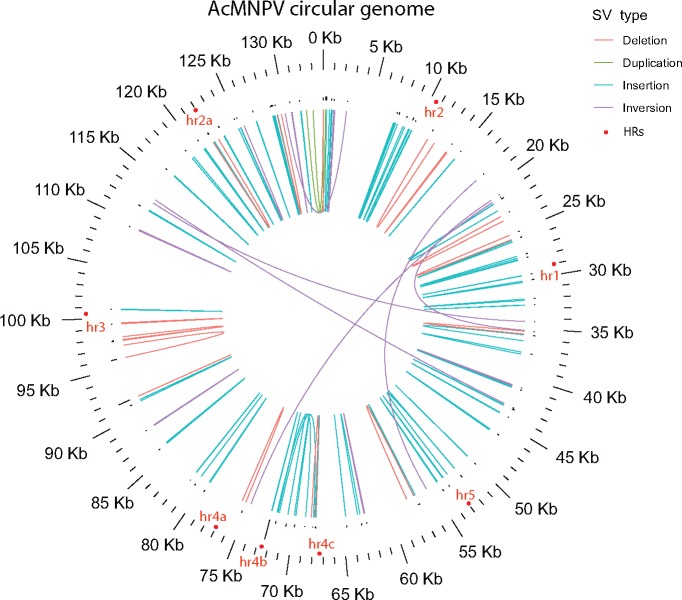
Map of the circular AcMNPV genome illustrating all SVs present in more than 0.1 per cent of the viral population sequenced with Illumina and PacBio technologies. Each SV is illustrated by a curve linking their start and end coordinates. Histograms on top of SVs correspond to the relative frequency of each SV, with the most frequent SV involving hr4b.

Our search for SVs in the AcMNPV short-read Illumina data using our clustering pipeline applied to the results of four SV callers (Lumpy, fermikit, pindel, and custom Python script) yielded 22,892 variants, among which 1,141 (4.98%) were considered robust as they overlapped with the 9,421 SVs detected in the PacBio long-read data. The 1,141 SVs comprised 464 deletions, 443 inversions, 160 duplications, and 74 insertions (Fig. 2A, Table 1 and Supplementary Table S1). Examples of read alignments supporting twelve AcMNPV SVs are shown in Supplementary Figs S2–S14. SV size ranged from 50 bp (the minimum size cutoff that we used) for an insertion to 66,787 bp for an inversion (close to the maximum size cutoff), with an average of 30,496 bp (Fig. 2B). SVs were detected all along the AcMNPV genome, with no apparent hotspot (Fig. 3 and Supplementary Fig. S15).

**Table 1.** Numbers, frequencies and lengths of SVs detected in AcMNPV, HCMV, IIV6, and IIV31 populations.

SV type	Number of SVs	Frequency (%)	Minimum length (bp)	Average length (bp)	Maximum length (bp)
AcMNPV					
DEL	464	11.91	52	29,318	66,712
DUP	160	2.05	290	38,898	66,488
INS	74	26.11	50	159	2,723
INV	443	5.70	55	33,762	66,787
Total	1,141	39.87	50	30,496	66,787
HCMV					
DEL	48	12.43	54	16,211	78,246
DUP	80	9.69	156	7,386	132,989
INS	0	0	0	0	0
INV	556	42.30	55	10,825	205, 218
Total	684	54.37	54	10,800	205, 218
IIV6					
DEL	346	47.16	52	10,060	207,988
DUP	113	8.90	171	21,386	180,808
INS	0	0	0	0	0
INV	643	1.14	105	61,168	206,392
Total	1,102	52.41	52	41,042	207,988
IIV31					
DEL	97	76.58	55	15,004	209,948
DUP	12	0.11	212	36,295	139,680
INS	7	6.72	58	63	66
INV	310	8.60	100	58,550	203,305
Total	426	80.06	55	47,046	209,948

The frequency refers to the percentage of viral genomes affected by the SV type, assuming that it follows a Poisson distribution. DEL, deletions; DUP, duplications; INS, insertions; INV, inversions. The frequencies were computed assuming the number of SVs per viral genome follows a Poisson distribution. Details about each SV detected in the four viral genomes are provided in Supplementary Tables S1, S4–S6.

**Figure 4.**
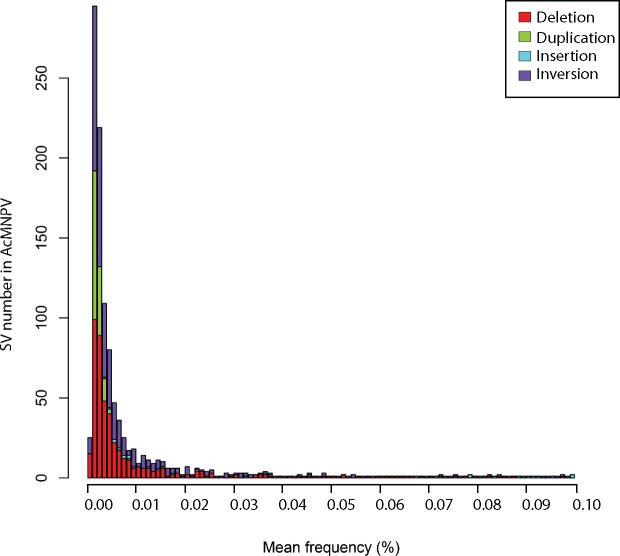
Number of SVs by 0.001 per cent frequency bin detected in the AcMNPV population sequenced using Illumina and PacBio technologies. Only the first 100 frequency bins are shown. The vast majority of SVs (92%) are present in viral genomes at a very low frequency (<0.01%).

Most AcMNPV SV variants occurred at low to very low frequencies, with 92.4 and 75.4 per cent of SVs having a frequency <0.1 and 0.01 per cent, respectively (Fig. 4). Yet, taking all 1,141 SVs into account and assuming that the number of SVs per viral genome follows a Poisson distribution, we calculated that no less than 39.9 per cent of AcMNPV genomes are affected by a variant (Supplementary Table S1). It is noteworthy that in spite of being less numerous than other SVs in the AcMNPV population, insertions were generally segregating at higher frequency, with an overall estimate of 26.1 per cent of AcMNPV genomes being affected by an insertion (Fig. 2C and Supplementary Table S1). The most frequent SV in this viral population was an insertion of ∼70 bp that increased the length of the hr4b homologous region in ∼6.0 per cent of AcMNPV genomes. Interestingly, the five most frequent SVs in this population involve intergenic regions, non-essential or uncharacterized genes (Supplementary Table S2), which may reflect the lower effect of these SVs on viral fitness. We also found that the total frequency of all SVs involving genes, hr, or intergenic regions was fairly homogeneous and mostly comprised between 1.9 and 6.2 per cent (Supplementary Fig. S16). The only exception to this pattern is the hr4b region mentioned above, which is involved in 535 SV affecting ∼10.8 per cent of viral genomes. Next, we counted the number of SVs involving each of the 151 AcMNPV genes and classified SVs as either inactivating (SVs inducing gene truncations) or non-inactivating (i.e. the coding capacity of the gene remains intact). We found that 148 out of the 151 genes were more affected by non-inactivating than by inactivating SVs. For these 148 genes, there was on average 55 inactivating and 100 non-inactivating SVs. Notably, the three remaining genes are located at the extremities of the linear AcMNPV genome as we have used it for the analyses. Thus the higher number of inactivating SVs in these genes is due to a technical effect. We also looked at the cumulative frequency of inactivating and non-inactivating SVs affecting genes. The results were consistent with the raw numbers of SVs, with the vast majority of genes (*N* = 149) more frequently affected by non-inactivating SVs than by inactivating SVs.

We then used the long-read dataset to assess the extent to which a given viral genome can be affected by multiple SVs. This analysis was based on the set of SV-carrying long reads detected by Sniffles only, as only this program provides read names in the output SV list. All 15,044 reads supporting the 1,648 SVs detected by Sniffles were retrieved. The vast majority of these reads (*N* = 14,783) carried a single SV, and the remaining 261 reads (1.74%) carried more than one SVs. Among these, only 13 and 1 reads, respectively carried three and four SVs. For 161 of the 261 reads carrying more than 1 SV, SV coordinates overlapped, indicating nested SVs.

### 3.3 Comparison of simulated and observed SVs

To assess the extent to which technical chimeras produced during the construction of the Illumina sequencing library may have introduced biases in SV count and frequency calculation, we generated a mock short-read dataset in which a proportion of chimeric reads were introduced (see Section 2.10). To compare the numbers, nature and frequency of SVs detected with this simulated dataset to the 1,141 robust SVs detected with the real dataset, we selected 4.98 per cent of all detected SVs using the simulated datasets (i.e. the proportion of SVs detected by both sequencing technologies among all SVs detected using short reads only, see above). The 4.98 per cent most frequent SVs supported by at least 2 SV callers were selected, which yielded 802 SVs, corresponding to 737 deletions and 65 duplications (Supplementary Table S3). Taking all these SVs into account, we calculated that 1.47 per cent of AcMNPV genomes carry one SV detected with the simulated dataset (assuming the number of SVs by viral genome follows a Poisson distribution). These results show that technical chimeras can induce a substantial number of false positives using our SV detection pipeline. However, the SV profile and frequency of viral genomes calculated to carry these variants widely differ between the simulated (737 deletions and 65 duplications; 1.47%) and real dataset (464 deletions, 443 inversions, 160 duplications, 74 insertions; 39.9%), strongly suggesting that the vast majority of SVs detected by both sequencing technologies in the real dataset are indeed biological. Note that the number of SVs due to technical chimeras that we detect in the mock dataset is likely overestimated because we chose to simulate reads with 5 per cent of chimeras, which corresponds to some of the highest technical chimera rates observed in previous studies (Görzer et al. 2010; Peccoud et al. 2018).

The construction of PacBio sequencing libraries, which involves a blunt-end ligation step, can also induce the formation of a substantial number of artefactual chimeras (Tallon et al. 2014; Griffith et al. 2018). However, the conditions and rates at which such chimeras are generated have been less studied than those produced during the construction of Illumina libraries. Currently available simulators of long PacBio reads do not offer the possibility to generate chimeras (Ono, Asai, and Hamada 2013; Stöcker, Köster, and Rahmann 2016; Wei and Zhang 2018; Zhang, Jia, and Wei 2019). Thus, we did not estimate the number of SVs possibly due to artificial long-read chimeras that we can detect with our pipeline.

### 3.4 Characterization of SVs in twenty-one AcMNPV datasets

**Figure 5.**
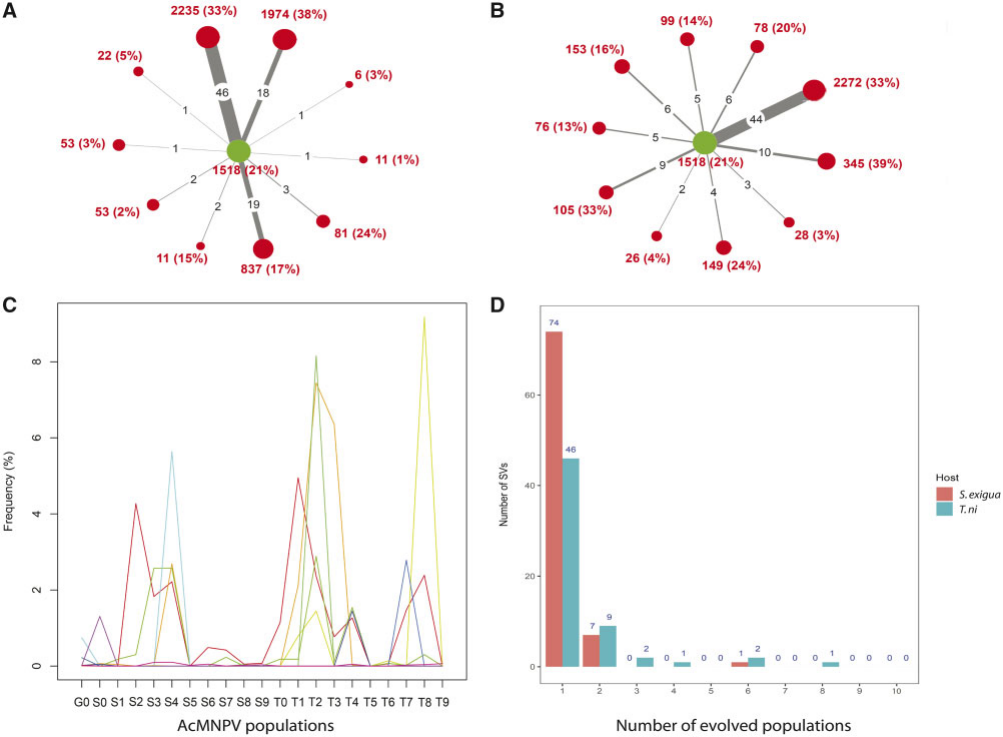
Dynamics of SVs in twenty evolved AcMNPV lines. (A) Red circles show the number of SVs detected in ten AcMNPV populations which were each purified after ten infection cycles on larvae of the beet armyworm (*S. exigua*). The green circle shows the number of SVs detected in the parental population of AcMNPV purified from larvae of the cabbage looper moth (*T. ni*). The size of the circle is proportional to the number of SVs and the frequency of viral genomes carrying a SV is given between brackets, assuming the number of SVs per viral genome follows a Poisson distribution. The thickness of the lines linking the parental AcMNPV population to each of the ten evolved populations is proportional to the number of shared SVs (numbers in black on the lines). (B) Same as in A except that the ten evolved AcMNPV populations were purified after ten infection cycles on larvae of the same species (*T. ni*) as that used to generate the parental AcMNPV population. (C) Frequency of the ten most frequent SVs detected among the twenty-one viral populations and which were initially present in the parental AcMNPV population. One color corresponds to one SV. The ‘G0’ population refers to the parental population. The S0–S9 populations refer to the populations evolved on *S. exigua* larvae. The T0–T9 populations refer to populations evolved on *T. ni*. Note that no SVs reached >10 per cent in frequency in any AcMNPV population. (D) Number of SVs present in the parental AcMNPV population that were also detected in one to ten evolved viral populations. Most SVs were only detected in one evolved population (seventy-four in *S. exigua* and forty-six in *T. ni*).

The finding of a large number of SVs in AcMNPV populations raised the question of their persistence over several rounds of infection. To investigate SV dynamics during viral evolution, we used a published experimental evolution dataset of AcMNPV, whereby a population of this virus purified after several rounds of infection on the cabbage looper moth (*T.* *ni*) was Illumina-sequenced at 187,536× average depth and was independently passaged in ten lines of *T. ni* larvae and ten lines of *S. exigua* larvae, each line consisting of ten successive infection cycles (see Gilbert et al. 2016 and Section 2). Overall, this analysis revealed that the number of SVs shared by the parental *T. ni* population and any of the twenty evolved populations was always low (from 1 [0.07%] to 46 [3.9%] out of the 1,158 SVs detected in the parental *T. ni* population; Fig. 5A and B), and that the vast majority of SVs were only present in one population (Fig. 5D). Of note, one SV present in the G0 population was found in eight *T. ni* datasets and in six *S. exigua* datasets. It is a duplication of 62,654 bp involving the hr2 and hr4b regions in very low frequency in the parental population (0.008%) that increased in frequency in some evolved populations (>4%, represented in red in Fig. 5C).

### 3.5 Analyses of SVs supported by short reads in populations of HCMV, IIV6, and IIV31

To assess the extent to which SVs may affect viruses other than AcMNPV, we generated short-read datasets for two invertebrate iridoviruses, IIV6, and IIV31, respectively passaged on caterpillars of the Mediterranean corn borer (*S. nonagrioides*) and the pillbug *A. vulgare*, and for human CMV passaged on MRC5 cells. The IIV6 genome we assembled was 210,812 bp in length, 99.51 per cent identical to and 1,670 bp shorter than the closest reference genome available in NCBI, that of the Chilo iridescent virus IIV6 (accession number AF303741.1). Over the 468 genes annotated in the reference IIV6 genome, 435 were recovered in our IIV6 assembly and used for downstream analyses. Among these 4thirty-five genes, twenty-two have a weak protein similarity (<60%) with those in the reference genome. The differences between our assembly and the reference genome were due to seventy-one insertions and fifty-four deletions, including twenty-one insertions and fifteen deletions located within genes without changing the open reading frame. Our assembly of the IIV31 genome was 219,807 bp in length, 99.90 per cent identical to and 415 bp shorter than the *A.* *vulgare* iridescent virus reference genome (accession number HF920637.1). A total of 193 out of 203 genes from the reference genome were retrieved in the assembly. The difference in length with the reference genome was due to thirty-seven insertions and forty-six deletions, among which eight insertions and eleven deletions were localized in open reading frames (without disruption). Finally, the HCMV genome we assembled was 234,915 bp in length, 97.69 per cent identical to and 731 bp shorter than its closest reference genome available in NCBI, that of the Merlin strain (accession number KP745639.1). A total of 151 genes out of 154 annotated in the Merlin strain were recovered in our assembly. The difference in length was due to ninety-two insertions and eighty-three deletions among which thirty-four insertions and twenty-two deletions affected open reading frames (without disruption).

The four variant callers used to identify AcMNPV SVs in short reads were run on the three additional viruses. As previously, we conservatively estimated the number of robust SVs as 4.98 per cent of the total number of SVs identified with the four variant callers. Following this approach, we counted a total of 684,426 and 1,102 SVs in HCMV, IIV31, and IIV6 datasets, respectively (Table 1 and Supplementary Tables S4–S6). We estimated that overall 54.4, 80.1, and 52.4 per cent of the HCMV, IIV31, and IIV6 viral genomes, respectively, were affected by SVs, assuming the number of SVs in viral genomes follows a Poisson distribution (Fig. 2). The two most abundant SV types affecting IIV31 and IIV6 genomes were deletions and inversions, while duplications and inversions were the main events occurring in the HCMV genomes (Fig. 2A). It is noteworthy that about 76.6 and 47.2 per cent of IIV31 and IIV6 genomes were affected by deletions, respectively. Furthermore, >42 per cent of HCMV genomes carried an inversion (Fig. 2C). SV sizes were very heterogeneous, ranging from 52 bp for a deletion in IIV6 genomes to 209,948 bp for a deletion in IIV31 genomes. The average mean size of SVs was 10,800, 47,046, and 41,042 bp in HCMV, IIV31, and IIV6, respectively (Fig. 2B). For all three viruses, SVs were detected all along the genome, suggesting no region was devoid of SVs (Supplementary Fig. S15). The five most frequent SVs in IIV6, IIV31, and HCMV populations mainly involve intergenic regions, non-essential or uncharacterized genes (Supplementary Table S2). Strikingly, the five most frequent SVs in the IIV6 population involve the 444 gene (unknown function) and account for >25 per cent of the SV frequency in the viral population.

### 3.6 TE insertions in viral genomes

**Figure 6.**
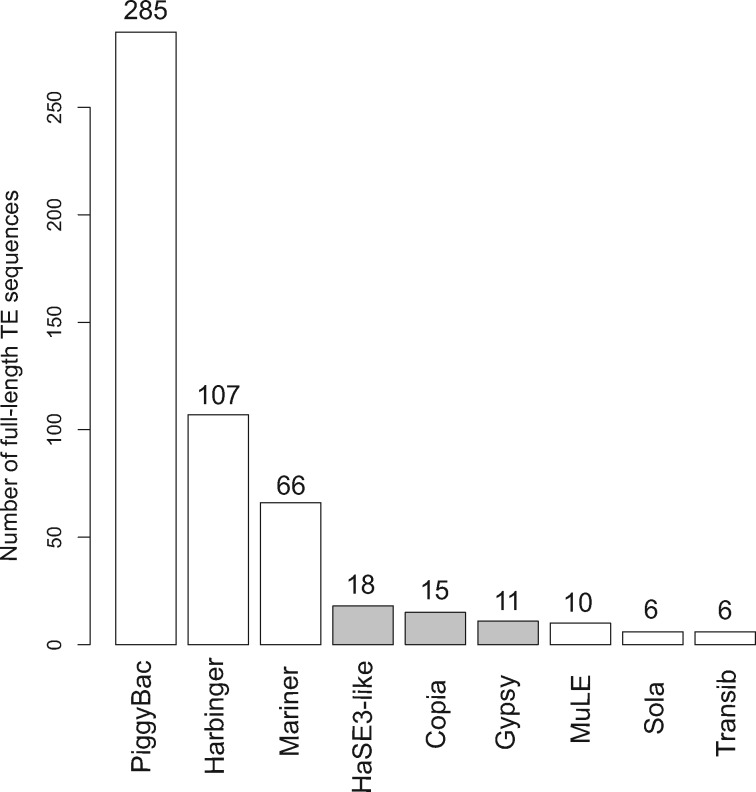
Number of TEs integrated as full-length copies for the nine TE superfamilies found in the AcMNPV genomes. Six and three TE superfamilies belong to Classes II and I TEs, respectively. The major part of full-length inserted TE sequences belong to the Class II TE superfamilies (480 complete TE sequences out of 524), mainly to the PiggyBac TE superfamily.

The seventy-four insertions detected in the AcMNPV population isolated from *S. exigua* and sequenced using both short- and long-read technologies all corresponded to insertions of AcMNPV sequences. These insertions could be considered duplications, but they were not classified as such by SV callers, presumably because duplicated sequences were not in tandem or located sufficiently close to each other along the AcMNPV genome. SV callers did not identify any insertion of non-AcMNPV DNA, which is somewhat surprising because our earlier works, based on twenty-one AcMNPV populations reanalyzed here, have shown that a large number of host TEs systematically integrate into AcMNPV genomes during infection (Gilbert et al. 2016). Using the same method as in Gilbert et al. (2016), we searched for host TEs in the short reads of the new AcMNPV population sequenced in this study. We identified 4,993 virus–host TE junctions involving one and nine superfamilies of Classes 1 and 2 TEs, respectively, and yielding an estimate of 1.5 per cent viral genomes harboring a host TE in this population (Table 2). Using the long-read dataset, we were further able to retrieve a total of 524 full-length TE copies from three Class 1 and six Class 2 TE superfamilies (Fig. 6). Another 1,233 TEs were identified in long reads as truncated copies. In contrast, no TE insertion was found using the Gilbert et al. (2016) pipeline in the HCMV, IIV6, and IIV31 genome populations.

**Table 2.** Characteristics of TE insertions detected in AcMNPV short-read data.

TE superfamily	Number of unique chimeric reads	Number of chimeric reads including PCR duplicates	Insertion frequency (%)
Sola	2,347	2,956	0.7
Harbinger	1,154	1,305	0.3
Gypsy	495	562	0.13
Mariner	361	1,078	0.24
piggyBac	349	398	0.09
Copia	180	319	0.07
Transib	51	57	0.01
MuLE	33	35	0.007
Helitron	22	22	0.005
hAT	1	10	0.002
Total	4,993	6,742	1.554

## 4. Discussion

Although SVs have been implicated as an important source of viral evolution in several large dsDNA viruses (López-Ferber et al. 2003; Elde et al. 2012; Mahiet et al. 2012; Chateigner et al. 2015; Filée, 2015; Karamitros et al. 2018; Sasani et al. 2018), the full spectrum and overall frequency of SVs carried by populations of these viruses has never been probed using high-throughput sequencing. Here, we begin tackling this issue by focusing on three invertebrate viruses for which obtaining large quantities of DNA from *in vivo* infections was possible (AcMNPV, IIV6, and IIV31) as well as on one human virus replicated in cell lines (HCMV). The rationale followed in this study to robustly estimate a minimum number of SVs segregating in populations of these large dsDNA viruses is that given that short- and long-read sequencing technologies are not affected by the same biases inducing the formation of technical chimeras (Tallon et al. 2014; Tsai et al., 2014; Griffith et al. 2018; Peccoud et al. 2018), SVs detected by both types of data can be considered biological. Although likely conservative, this approach revealed that populations of AcMNPV can carry more than one thousand SVs together affecting almost 40 per cent of genomes. Based on the proportion of AcMNPV SVs detected by both sequencing technologies among all SVs detected by Illumina sequencing only (4.98%) we have estimated that much like in AcMNPV, several hundreds to more than 1,000 SVs can be found in populations of three other large dsDNA viruses (HCMV, IIV6, and IIV31). The number of SVs found in these dsDNA viruses is thus similar to those circulating in RNA virus populations (Jaworski and Routh 2017). Our results are in agreement with earlier molecular biology studies (Mahiet et al. 2012) and further contribute to unveil SVs as an important facet of the biology of large dsDNA viruses.

The large number of programs that have been developed to detect SVs in short- and long-sequencing reads all rely on different approaches involving different algorithms and/or are based on different mapping strategies. Integrating the results of these various programs to detect as many robust SVs as possible was a challenge. While some meta-callers pooling the results of several SV callers exist (Wong et al. 2010; Mohiyuddin et al. 2015; Zarate et al. 2018), they were all geared to SVs detection in gigabase-sized genomes sequenced at <100×, conditions that vastly differ from our study of kilobase-sized genomes sequenced at depth ranging from >3,000 to >200,000×. We thus developed our own meta-calling approach based on hierarchical clustering of SVs detected by only some of the programs that are available. Our choice of programs was guided by limitations of some of these programs to effectively analyze our data. For example, Manta (Chen et al. 2016), Delly (Rausch et al. 2012), and Wham (Kronenberg et al. 2015) were unable to detect more than thirty SVs in the AcMNPV Illumina datasets, likely because they were benchmarked on data corresponding to <50× sequencing depth or they automated the exclusion of deeply covered regions (Kronenberg et al. 2015; Chen et al. 2016). On a related methodological note, we further monitored the influence sequencing depth has on the detection of SVs by subsampling our >200,000× AcMNPV Illumina dataset at depths ranging from 50 to 50,000× and running our SV detection pipeline on these subsamples. We found that sequencing depth had a strong impact on the number of detected SVs, with <150 SVs detected at depths ≤50,000× compared with the 1,141 SVs detected at >200,000× (Supplementary Fig. S17). Thus, a large fraction of low frequency variants segregating in viral populations cannot be detected with our approach unless extremely high-sequencing depths are generated.

SVs have never been characterized in IIV6 and IIV31 so we cannot compare the nature of the SVs detected in this study to previous studies. In regard to HCMV, Karamitros et al. (2018) performed long-read sequencing of the TB40/E strain, which enabled precise characterization of a 1,348-bp deletion located between genes UL144 and UL145. This SV was not identified in our HCMV short-read data; instead we found a deletion of a 350-bp intergenic region located between these same two genes with a frequency of 0.09 per cent. The two SVs are different, but they show that this genomic region is susceptible to deletions in both strains. Regarding AcMNPV, Chateigner et al. (2015) characterized short deletions in a population of this virus using short-read sequencing. The majority of deletions were found to involve hr1, hr2, hr3, and hr4b, which are homologous regions scattered around the AcMNPV genome thought to serve as origin of replication (Pearson et al. 1992; Kool et al. 1995). Here, we found 114 AcMNPV SVs involving hr regions in the population sequenced by both Illumina and PacBio technologies. We calculated that seven per cent of AcMNPV genomes harbor one SV involving an hr. These SVs correspond to 103 deletions, six inversions, three duplications, and two insertions. The hr regions most frequently involved in SVs are hr2, hr3, hr4b, and hr5, which is concordant with Chateigner et al. (2015) and further highlights the role of AcMNPV hr regions in producing SVs.

The fact that the majority of SVs are present at low to very low frequencies in all viral populations indicates they are likely deleterious and thus unlikely to persist over many rounds of replication. Accordingly, we found a higher frequency of non-inactivating versus inactivating SVs in most AcMNPV genes and very few SVs were shared between the parental *T. ni* AcMNPV population and the twenty populations of this virus that underwent ten infection cycles on *T. ni* or *S. exigua*. These findings echo the low number of TE insertions we found to be shared between the parental *T. ni* and the twenty evolved AcMNPV populations in our earlier study (Gilbert et al. 2016) and indicate that much like TE insertions, other SVs are continuously gained and lost at high rates during viral replication. This rapid SV turnover likely involve recombination (Crouch and Passarelli 2002; Kamita, Maeda, and Hammock 2003; Sijmons et al., 2015; Cudini et al. 2019) as well as errors in viral genome replication (Kilpatrick and Huang 1977; van Oers and Vlak 2007) and DNA repair (Kulkarni and Fortunato 2011; Xiaofei and Kowalik 2014; Renzette et al. 2015). It would be interesting to assess the relative importance of each of these mechanisms in generating structural diversity in the future.

It has been proposed that large SVs, including gene captures, gene duplications, and deletions play a crucial role in the adaptation of large dsDNA viruses to new hosts (Elde et al. 2012; Filée 2013; Thézé et al. 2015; Sasani et al. 2018). Our study was not designed to assess the adaptive role of SVs but it is noteworthy that the experiment producing the twenty-one AcMNPV short-read datasets involved a host switch from *T. ni* to *S. exigua* larvae. In this context, an independent increase in the frequency of a given SV in several AcMNPV lines replicated on *S. exigua* (coupled to no increase of the same SV in AcMNPV lines replicated on *T. ni*) would have provided an indication that this SV might have been involved in adaptation. Our analysis did not reveal any evidence of such a situation, nor did it reveal any case of polymorphic insertion involving a host gene. One reason for the absence of detectable adaptive AcMNPV SV after switching the virus from *T. ni* to *S. exigua* might be that in spite of diverging by more than 60 Myrs (Toussaint et al. 2012) the two noctuids used to generate those lines are too closely related to expect any major viral adaptation during a switch from one host to the other. Another possibility is that large SVs such as those on which we focused in this study are in fact rarely involved in viral adaptation because their effects on viral replication are too large. Interestingly, close inspection and comparison of the newly assembled consensus genome of the four viruses with the closest genomes available in GenBank revealed a number of differences involving small (<50 bp) variants. Since the GenBank viruses most closely related to AcMNPV, HCMV, and IIV6 were sequenced from different hosts (Sf9 cells for AcMNPV, E_1_SM fibroblasts for HCMV, and CF-124 cells for IIV6) compared with this study, it is possible that the change in frequency of these small variants are due to their effect on the viral fitness in the different hosts. Interestingly, small variants are also found between our IIV31 genome and its closest relative in GenBank even though both viruses were isolated from *A. vulgare* (Piegu et al. 2014). These differences could be due to virus adaptation to different genetic backgrounds in pillbug populations or neutral genomic changes. Small variants may be more often involved in adaptation to host switches than larger ones because of their smaller effect on viral replication. Yet, it is also possible that several of these variants have no effect on viral fitness and became fixed through drift.

The short-read sequencing of a new AcMNPV population purified from *S. exigua* confirms our earlier observation of thousands of host TEs integrated in AcMNPV genomes (Gilbert et al. 2016). One limitation of short reads to analyze host TEs integrated into viral genomes is that it is impossible to assess whether reads mapping entirely on TEs originate from TE copies integrated into the virus genome or from copies integrated into contaminating fragments of the host genome. Thus, the completeness of TE copies integrated into AcMNPV genomes cannot be assessed using short reads. In agreement with previous low-throughput approaches (Fraser et al., 1995; Jehle et al. 1995, 1997), our long-read sequencing data shows that within an AcMNPV population, hundreds of TEs are integrated into AcMNPV genomes as full length copies. Although the high error rate of PacBio sequencing does not allow assessment of whether these TE copies are free from non-sense mutations, such high numbers of full length copies suggest that many of these TE are functional and potentially able to further jump from the viral genome into another genome, which may be that of another host infected by AcMNPV. Thus, our results further support the role of AcMNPV as potential vector of horizontal transfer of TEs between insects (Miller and Miller, 1982; Gilbert et al. 2014, 2016; Gilbert and Cordaux 2017).

The finding of many TEs integrated into AcMNPV genomes contrasts with the absence of TEs in all consensus baculovirus genomes sequenced so far, which suggests that TE insertions never reach high frequencies in viral populations (Gilbert et al. 2016). Thus, though the rate of TE transfer from host to virus is relatively high, the probability of TEs to have either a positive or no impacts on the virus fitness, and thus to increase in frequency in a viral population, is extremely low. The absence of polymorphic host gene insertions in AcMNPV populations surprisingly contrasts with the relatively large number of host genes that have been captured by baculoviruses during their evolution (Hughes and Friedman 2003; Thézé et al. 2015). Thus, contrary to TEs, while host genes may rarely end up integrated into baculovirus genomes, their chances to improve viral fitness may be much higher than that of TEs.

The finding of many truncated TE copies in AcMNPV long reads is also interesting considering that the majority (895 out of 1,233) of these truncated copies begin or terminate the read in which they were found that is, they are flanked by viral sequences only at one of their ends. The high number of truncated TEs at the extremities of long reads does not correspond to what would be expected if truncated TEs were randomly distributed in long reads (exact binomial test, *P*-value < 2.2*10^−^^16^, see Section 2). It is thus possible that at least a subset of truncated TE copies at read extremities correspond to the very extremity of linear AcMNPV genome molecules. In turn, such linear AcMNPV genomes could result from aborted transposition that led to the formation of truncated TE copies. Interestingly, linearized AcMNPV genomes are known to be 15- to 150-fold less infectious than circular forms (Kitts, Ayres, and Possee 1990). Thus, linearization of AcMNPV genomes mediated by aborted transposition could be viewed as beneficial by-product of transposition, which may impede or slow down AcMNPV replication. Here, the number of potentially linear AcMNPV genomes containing truncated TE copies is relatively low compared with TE-free genomes in the population we sequenced. Thus, the potential impact transposition-induced linearization may have on AcMNPV replication is unlikely to be significant in *S. exigua*. Yet, the possible antiviral protection conferred by aborted transposition of host TEs may be viewed as a form of cooperation between TEs and their hosts (Cosby, Chang, and Feschotte 2019) and worthy of further investigation in other host/baculovirus systems.

Finally, the absence of TE copies integrated in HCMV, IIV6, and IIV31 contrasts with their widespread occurrence in AcMNPV. It may be explained either by a low TE activity in human MRC5 cells, *S. nonagrioides* and *A. vulgare*, and/or by a weak capacity for the virus to carry supplementary genomic loads like TEs. This observation also contributes to make AcMNPV a better carrier of host TEs than other large dsDNA viruses, which, combined with its specificity for lepidopterans, may in part explain the higher number of horizontal transfer of TEs recently inferred in these compared with other arthropods (Reiss et al. 2019).

## Data availability

The various sequencing datasets produced during this study have been deposited in the SRA database of the NCBI under accession number PRJNA592818. All supplementary data, figures, tables and R scripts associated to this manuscript have been deposited in the DRYAD database (datadryad.org): DOI https://doi.org/10.5061/dryad.cfxpnvx25. This includes fasta files of the newly assembled AcMNPV, HCMV, IIV6 and IIV31 genomes, as well as their annotation files.

**Conflict of interest:** None declared.

## Funding

This work was supported by Agence Nationale de la Recherche Grant ANR-15-CE32-0011-01 TransVir (to C.G.)

## Supplementary Material

vez060_Supplementary_Data

## References

[vez060-B1] Acevedo A., Andino R. (2014) ‘Library Preparation for Highly Accurate Population Sequencing of RNA Viruses’, Nature Protocols, 9: 1760–9. 24967624 10.1038/nprot.2014.118 PMC4418788

[vez060-B2] Acevedo A., Brodsky L., Andino R. (2014) ‘Mutational and Fitness Landscapes of an RNA Virus Revealed through Population Sequencing’, Nature, 505: 686–90. 24284629 10.1038/nature12861 PMC4111796

[vez060-B3] Ackermann H.-W., Smirnoff W. A. (1983) ‘A Morphological Investigation of 23 Baculoviruses’, Journal of Invertebrate Pathology, 41: 269–80.

[vez060-B4] Akhtar L. N. et al (2019) ‘Genotypic and Phenotypic Diversity of Herpes Simplex Virus 2 within the Infected Neonatal Population’, MSphere, 4: e00590–18 10.1128/mSphere.00590-18 PMC6393728 30814317

[vez060-B5] Alkan C., Coe B. P., Eichler E. E. (2011) ‘Genome Structural Variation Discovery and Genotyping’, Nature Reviews Genetics, 12: 363–76. 10.1038/nrg2958 PMC4108431 21358748

[vez060-B6] Angly F. E. et al (2012) ‘Grinder: A Versatile Amplicon and Shotgun Sequence Simulator’, Nucleic Acids Research, 40: e94. 22434876 10.1093/nar/gks251 PMC3384353

[vez060-B7] Bankevich A. et al (2012) ‘SPAdes: A New Genome Assembly Algorithm and Its Applications to Single-Cell Sequencing’, Journal of Computational Biology, 19: 455–77. 22506599 10.1089/cmb.2012.0021 PMC3342519

[vez060-B8] Bao W., Kojima K. K., Kohany O. (2015) ‘Repbase Update, a Database of Repetitive Elements in Eukaryotic Genomes’, Mobile DNA, 6:10.1186/s13100-015-0041-9 PMC4455052 26045719

[vez060-B9] Benson D. A. et al (2005) ‘GenBank’, Nucleic Acids Research, 33: D34–D38. 15608212 10.1093/nar/gki063 PMC540017

[vez060-B10] Bull J. C., Godfray H. C. J., O'Reilly D. R. (2003) ‘A Few-Polyhedra Mutant and Wild-Type Nucleopolyhedrovirus Remain as a Stable Polymorphism during Serial Coinfection in *Trichoplusia ni**’*, Applied and Environmental Microbiology, 69: 2052–7. 12676682 10.1128/AEM.69.4.2052-2057.2003 PMC154768

[vez060-B11] Chaisson M. J., Tesler G. (2012) ‘Mapping Single Molecule Sequencing Reads Using Basic Local Alignment with Successive Refinement (BLASR): Application and Theory’, BMC Bioinformatics, 13:238 22988817 10.1186/1471-2105-13-238 PMC3572422

[vez060-B12] Chateigner A. et al (2015) ‘Ultra Deep Sequencing of a Baculovirus Population Reveals Widespread Genomic Variations’, Viruses, 7: 3625–46. 26198241 10.3390/v7072788 PMC4517117

[vez060-B13] Chebbi M. A. et al (2019) ‘The Genome of *Armadillidium vulgare* (Crustacea, Isopoda) Provides Insights into Sex Chromosome Evolution in the Context of Cytoplasmic Sex Determination’, Molecular Biology and Evolution, 36: 727–41. 30668787 10.1093/molbev/msz010

[vez060-B14] Chen X. et al (2016) ‘Manta: Rapid Detection of Structural Variants and Indels for Germline and Cancer Sequencing Applications’, Bioinformatics, 32: 1220–2. 26647377 10.1093/bioinformatics/btv710

[vez060-B15] Cosby R. L., Chang N.-C., Feschotte C. (2019) ‘Host–Transposon Interactions: Conflict, Cooperation, and Cooption’, Genes & Development, 33: 1098–116. 31481535 10.1101/gad.327312.119 PMC6719617

[vez060-B03978320] Craig N. L. (2002) ‘Mobile DNA: An Introducton', in Craig, N. L., Craig, R., Gellert, M., and Lambowitz A. M. (eds.) Mobile DNA II, pp. 3–11. Washington DC: ASM Press.

[vez060-B16] Crouch E. A., Passarelli A. L. (2002) ‘Genetic Requirements for Homologous Recombination in *Autographa californica* Nucleopolyhedrovirus’, Journal of Virology, 76: 9323–34. 12186915 10.1128/JVI.76.18.9323-9334.2002 PMC136457

[vez060-B17] Cudini J. et al (2019) ‘Human Cytomegalovirus Haplotype Reconstruction Reveals High Diversity Due to Superinfection and Evidence of Within-Host Recombination’, Proceedings of the National Academy of Sciences of the United States of America, 116: 5693–8. 30819890 10.1073/pnas.1818130116 PMC6431178

[vez060-B18] De Gooijer C. D. et al (1992) ‘A Structured Dynamic Model for the Baculovirus Infection Process in Insect-Cell Reactor Configurations’, Biotechnology and Bioengineering, 40: 537–48. 18601149 10.1002/bit.260400413

[vez060-B19] Duffy S., Shackelton L. A., Holmes E. C. (2008) ‘Rates of Evolutionary Change in Viruses: Patterns and Determinants’, Nature Reviews Genetics, 9: 267–76. 10.1038/nrg2323 18319742

[vez060-B20] Elde N. C. et al (2012) ‘Poxviruses Deploy Genomic Accordions to Adapt Rapidly against Host Antiviral Defenses’, Cell, 150: 831–41. 22901812 10.1016/j.cell.2012.05.049 PMC3499626

[vez060-B21] English A. C., Salerno W. J., Reid J. G. (2014) ‘PBHoney: Identifying Genomic Variants via Long-Read Discordance and Interrupted Mapping’, BMC Bioinformatics, 15: 180. 24915764 10.1186/1471-2105-15-180 PMC4082283

[vez060-B22] Filée J. (2013) ‘Route of NCLDV Evolution: The Genomic Accordion’, Current Opinion in Virology, 3: 595–9. 23896278 10.1016/j.coviro.2013.07.003

[vez060-B23] Filée J. (2015) ‘Genomic Comparison of Closely Related Giant Viruses Supports an Accordion-like Model of Evolution’, Frontiers in Microbiology, 6: 593. 26136734 10.3389/fmicb.2015.00593 PMC4468942

[vez060-B24] Fraser M. J. et al (1995) ‘Assay for Movement of Lepidopteran Transposon IFP2 in Insect Cells Using a Baculovirus Genome as a Target DNA’, Virology, 211: 397–407. 7645244 10.1006/viro.1995.1422

[vez060-B9496160] Fukaya M., Nasu S. (1966) ‘A Chilo Iridescent Virus (CIV) from the Rice Stem Borer, Chilo Suppressalis WALKER (Lepidoptera : Pyralidae)’, Applied Entomology and Zoology, 1: 69–72.

[vez060-B25] Gilbert C., Cordaux R. (2017) ‘Viruses as Vectors of Horizontal Transfer of Genetic Material in Eukaryotes’, Current Opinion in Virology, 25: 16–22. 28672159 10.1016/j.coviro.2017.06.005

[vez060-B26] Gilbert C. et al (2014) ‘Population Genomics Supports Baculoviruses as Vectors of Horizontal Transfer of Insect Transposons’, Nature Communications, 5: 3348. 10.1038/ncomms4348 PMC3948050 24556639

[vez060-B27] Gilbert C. et al (2016) ‘Continuous Influx of Genetic Material from Host to Virus Populations’, PLoS Genetics, 12: e1005838. 26829124 10.1371/journal.pgen.1005838 PMC4735498

[vez060-B28] Godfray H. C. J., Reilly D. R. O., Briggs C. J. (1997) ‘A Model of Nucleopolyhedrovirus (NPV) Population Genetics Applied to Co-occlusion and the Spread of the Few Polyhedra (FP) Phenotype’, Proceedings of the Royal Society of London. Series B: Biological Sciences, 264: 315–22.

[vez060-B29] Görzer I. et al (2010) ‘The Impact of PCR-Generated Recombination on Diversity Estimation of Mixed Viral Populations by Deep Sequencing’, Journal of Virological Methods, 169: 248–52. 20691210 10.1016/j.jviromet.2010.07.040

[vez060-B30] Griffith P. et al (2018). ‘PacBio Library Preparation Using Blunt-End Adapter Ligation Produces Significant Artefactual Fusion DNA Sequences’. BioRxiv.

[vez060-B31] Hughes A. L., Friedman R. (2003) ‘Genome-Wide Survey for Genes Horizontally Transferred from Cellular Organisms to Baculoviruses’, Molecular Biology and Evolution, 20: 979–87. 12716988 10.1093/molbev/msg107

[vez060-B25264775] Jain A. K. and Dubes R. C. (1988) Algorithms for Clustering Data, Prentice-Hall advanced reference series. Upper Saddle River, NJ: Prentice-Hall Inc.

[vez060-B32] Jaworski E., Routh A. (2017) ‘Parallel ClickSeq and Nanopore Sequencing Elucidates the Rapid Evolution of Defective-Interfering RNAs in Flock House Virus’, PLoS Pathogens, 13: e1006365. 28475646 10.1371/journal.ppat.1006365 PMC5435362

[vez060-B33] Jehle J. A. et al (1995) ‘TCl4.7: A Novel Lepidopteran Transposon Found in Cydia Pomonella Granulosis Virus’, Virology, 207: 369–79. 7886941 10.1006/viro.1995.1096

[vez060-B34] Jehle J. A. et al (1997) ‘Identification and Sequence Analysis of the Integration Site of Transposon TCp3.2 in the Gneome of Cydia Pomonella Granulovirus’, Virus Research, 50: 151–7. 9282780 10.1016/s0168-1702(97)00066-x

[vez060-B35] Kamita S. G., Maeda S., Hammock B. D. (2003) ‘High-Frequency Homologous Recombination between Baculoviruses Involves DNA Replication’, Journal of Virology, 77: 13053–61. 14645562 10.1128/JVI.77.24.13053-13061.2003 PMC296086

[vez060-B36] Karamitros T. et al (2016) ‘De Novo Assembly of Human Herpes Virus Type 1 (HHV-1) Genome, Mining of Non-Canonical Structures and Detection of Novel Drug-Resistance Mutations Using Short- and Long-Read Next Generation Sequencing Technologies’, PLoS One, 11: e0157600. 27309375 10.1371/journal.pone.0157600 PMC4910999

[vez060-B37] Karamitros T. et al (2018) ‘Nanopore Sequencing and Full Genome de Novo Assembly of Human Cytomegalovirus TB40/E Reveals Clonal Diversity and Structural Variations’, BMC Genomics, 19: 577. 30068288 10.1186/s12864-018-4949-6 PMC6090854

[vez060-B38] Kilpatrick B. A., Huang E.-S. (1977) ‘Human Cytomegalovirus Genome: Partial Denaturation Map and Organization of Genome Sequences’, Journal of Virology, 24: 16. 10.1128/jvi.24.1.261-276.1977 PMC515928 198578

[vez060-B39] Kitts P. A., Ayres M. D., Possee R. D. (1990) ‘Linearization of Baculovirus DNA Enhances the Recovery of Recombinant Virus Expression Vectors’, Nucleic Acids Research, 18: 5667–72. 2216760 10.1093/nar/18.19.5667 PMC332298

[vez060-B40] Kool M. et al (1991) ‘Detection and Analysis of *Autographa californica* Nuclear Polyhedrosis Virus Mutants with Defective Interfering Properties’, Virology, 183: 739–46. 1853572 10.1016/0042-6822(91)91003-y

[vez060-B41] Kool M. et al (1995) ‘Replication of Baculovirus DNA’, Journal of General Virology, 76: 2103–18. 7561748 10.1099/0022-1317-76-9-2103

[vez060-B42] Koren S. et al (2017) ‘Canu: Scalable and Accurate Long-Read Assembly via Adaptive *k*-Mer Weighting and Repeat Separation’, Genome Research, 27: 722–36. 28298431 10.1101/gr.215087.116 PMC5411767

[vez060-B43] Kronenberg Z. N. et al (2015) ‘Wham: Identifying Structural Variants of Biological Consequence’, PLoS Computational Biology, 11: e1004572. 26625158 10.1371/journal.pcbi.1004572 PMC4666669

[vez060-B44] Kulkarni A. S., Fortunato E. A. (2011) ‘Stimulation of Homology-Directed Repair at I-SceI-Induced DNA Breaks during the Permissive Life Cycle of Human Cytomegalovirus’, Journal of Virology, 85: 6049–54. 21490102 10.1128/JVI.02514-10 PMC3126324

[vez060-B45] Lauring A. S., Andino R. (2010) ‘Quasispecies Theory and the Behavior of RNA Viruses’, PLoS Pathogens, 6: e1001005. 20661479 10.1371/journal.ppat.1001005 PMC2908548

[vez060-B46] Lauring A. S., Frydman J., Andino R. (2013) ‘The Role of Mutational Robustness in RNA Virus Evolution’, Nature Reviews Microbiology, 11: 327–36. 23524517 10.1038/nrmicro3003 PMC3981611

[vez060-B47] Layer R. M. et al (2014) ‘LUMPY: A Probabilistic Framework for Structural Variant Discovery’, Genome Biology, 15: R84. 24970577 10.1186/gb-2014-15-6-r84 PMC4197822

[vez060-B48] Li H. (2015) ‘FermiKit: Assembly-Based Variant Calling for Illumina Resequencing Data’, Bioinformatics, 31: 3694-3696. 26220959 10.1093/bioinformatics/btv440 PMC4757955

[vez060-B49] Li H., Durbin R. (2009) ‘Fast and Accurate Short Read Alignment with Burrows-Wheeler Transform’, Bioinformatics, 25: 1754–60. 19451168 10.1093/bioinformatics/btp324 PMC2705234

[vez060-B50] Li D. et al (2011) ‘Defective Interfering Viral Particles in Acute Dengue Infections’, PLoS One, 6: e19447. 21559384 10.1371/journal.pone.0019447 PMC3084866

[vez060-B51] Lopez C. B. (2014) ‘Defective Viral Genomes: Critical Danger Signals of Viral Infections’, Journal of Virology, 88: 8720–3. 24872580 10.1128/JVI.00707-14 PMC4136278

[vez060-B52] López-Ferber M. et al (2003) ‘Defective or Effective? Mutualistic Interactions between Virus Genotypes’, Proceedings of the Royal Society of London. Series B: Biological Sciences, 270: 2249–55. 14613611 10.1098/rspb.2003.2498 PMC1691503

[vez060-B53] Lupetti P. et al (2013) ‘Iridovirus Infection in Terrestrial Isopods from Sicily (Italy)’, Tissue and Cell, 45: 321–7. 23756498 10.1016/j.tice.2013.05.001

[vez060-B54] Maghodia A. B., Jarvis D. L., Geisler C. (2014) ‘Complete Genome Sequence of the *Autographa californica* Multiple Nucleopolyhedrovirus Strain E2’, Genome Announc, 2: e01202−14. 10.1128/genomeA.01202-14 PMC4263824 25502662

[vez060-B55] Mahiet C. et al (2012) ‘Structural Variability of the Herpes Simplex Virus 1 Genome *in Vitro* and *in Vivo*’, Journal of Virology, 86: 8592–601. 22674981 10.1128/JVI.00223-12 PMC3421737

[vez060-B56] Manzoni T. B., López C. B. (2018) ‘Defective (Interfering) Viral Genomes Re-Explored: Impact on Antiviral Immunity and Virus Persistence’, Future Virology, 13: 493–503. 30245734 10.2217/fvl-2018-0021 PMC6136085

[vez060-B57] Marriott A. C., Dimmock N. J. (2010) ‘Defective Interfering Viruses and Their Potential as Antiviral Agents’, Reviews in Medical Virology, 20: 51–62. 20041441 10.1002/rmv.641

[vez060-B0488051] Miller D. W., Miller L. K. (1982) ‘A Virus Mutant with an Insertion of a Copia-like Transposable Element’, *Nature*, 299: 562–4. 6289125 10.1038/299562a0

[vez060-B58] Mohiyuddin M. et al (2015) ‘MetaSV: An Accurate and Integrative Structural-Variant Caller for Next Generation Sequencing’, Bioinformatics, 31: 2741–4. 25861968 10.1093/bioinformatics/btv204 PMC4528635

[vez060-B59] Mönchgesang S. et al (2016) ‘Natural Variation of Root Exudates in *Arabidopsis thaliana*-Linking Metabolomic and Genomic Data’, Science Reports, 6: 1–11. 10.1038/srep29033 PMC4929559 27363486

[vez060-B60] Müllner D. (2013) ‘Fastcluster: Fast Hierarchical, Agglomerative Clustering Routines for *R* and *Python*’, Journal of Statistical Software, 53: 1–18.

[vez060-B61] O’Reilly D. R., Miller L. K., Luckow V. A. (1992). Baculovirus Expression Vectors, A Laboratory Manual. New York: W.H. Freeman and Co.

[vez060-B62] Ono Y., Asai K., Hamada M. (2013) ‘PBSIM: PacBio Reads Simulator—Toward Accurate Genome Assembly’, Bioinformatics, 29: 119–21. 23129296 10.1093/bioinformatics/bts649

[vez060-B63] Parikh H. et al (2016) ‘Svclassify: A Method to Establish Benchmark Structural Variant Calls’, BMC Genomics, 17:64. 26772178 10.1186/s12864-016-2366-2 PMC4715349

[vez060-B64] Pearson M. et al (1992) ‘The *Autographa californica* Baculovirus Genome: Evidence for Multiple Replication Origins’, Science, 257: 1382–4. 1529337 10.1126/science.1529337

[vez060-B65] Peccoud J. et al (2017) ‘Massive Horizontal Transfer of Transposable Elements in Insects’, Proceedings of the National Academy of Sciences of the United States of America, 114: 4721–6. 28416702 10.1073/pnas.1621178114 PMC5422770

[vez060-B66] Peccoud J. et al (2018) ‘A Survey of Virus Recombination Uncovers Canonical Features of Artificial Chimeras Generated during Deep Sequencing Library Preparation’, G3 Genes Genomes Genetics, 8: 1129–38. 29434031 10.1534/g3.117.300468 PMC5873904

[vez060-B67] Piegu B. et al (2014) ‘Genome Sequence of a Crustacean Iridovirus, IIV31, Isolated from the Pill Bug, *Armadillidium vulgare*’, Journal of General Virology, 95: 1585–90. 24722681 10.1099/vir.0.066076-0

[vez060-B68] Poirier E. Z. et al (2018) ‘Dicer-2-Dependent Generation of Viral DNA from Defective Genomes of RNA Viruses Modulates Antiviral Immunity in Insects’, Cell Host & Microbe, 23: 353–65.e8. 29503180 10.1016/j.chom.2018.02.001 PMC5857290

[vez060-B69] Quinlan A. R., Hall I. M. (2010) ‘BEDTools: A Flexible Suite of Utilities for Comparing Genomic Features’, Bioinformatics, 26: 841–2. 20110278 10.1093/bioinformatics/btq033 PMC2832824

[vez060-B70] Rausch T. et al (2012) ‘DELLY: Structural Variant Discovery by Integrated Paired-End and Split-Read Analysis’, Bioinformatics, 28: i333–i339. 22962449 10.1093/bioinformatics/bts378 PMC3436805

[vez060-B9038681] R Core Team. (2018) R: A Language and Environment for Statistical Computing. Vienna: R Foundation for Statistical Computing.

[vez060-B71] Reiss D. et al (2019) ‘Global Survey of Mobile DNA Horizontal Transfer in Arthropods Reveals Lepidoptera as a Prime Hotspot’, PLoS Genetics, 15: e1007965. 30707693 10.1371/journal.pgen.1007965 PMC6373975

[vez060-B72] Renzette N. et al (2013) ‘Rapid Intrahost Evolution of Human Cytomegalovirus is Shaped by Demography and Positive Selection’, PLoS Genetics, 9: e1003735. 24086142 10.1371/journal.pgen.1003735 PMC3784496

[vez060-B73] Renzette N. et al (2015) ‘Limits and Patterns of Cytomegalovirus Genomic Diversity in Humans’, Proceedings of the National Academy of Sciences of the United States of America, 112: E4120–8. 26150505 10.1073/pnas.1501880112 PMC4522815

[vez060-B74] Renzette N. et al (2017) ‘On the Analysis of Intrahost and Interhost Viral Populations: Human Cytomegalovirus as a Case Study of Pitfalls and Expectations’, Journal of Virology, 91: e01976–16. 27974561 10.1128/JVI.01976-16 PMC5309957

[vez060-B75] Rohrmann G. F. (2014) ‘Baculovirus Nucleocapsid Aggregation (MNPV vs SNPV): an Evolutionary Strategy, or a Product of Replication Conditions? ’, Virus Genes, 49: 351–7. 25224849 10.1007/s11262-014-1113-5

[vez060-B76] Routh A. et al (2015) ‘ClickSeq: Fragmentation-Free Next-Generation Sequencing via Click Ligation of Adaptors to Stochastically Terminated 3′-Azido cDNAs’, Journal of Molecular Biology, 427: 2610–6. 26116762 10.1016/j.jmb.2015.06.011 PMC4523409

[vez060-B77] Sanjuán R., Domingo-Calap P. (2016) ‘Mechanisms of Viral Mutation’, Cellular and Molecular Life Sciences, 73: 4433–48. 27392606 10.1007/s00018-016-2299-6 PMC5075021

[vez060-B78] Sasani T. A. et al (2018) ‘Long Read Sequencing Reveals Poxvirus Evolution through Rapid Homogenization of Gene Arrays’, Elife, 7: e35453. 10.7554/eLife.35453 PMC6115191 30156554

[vez060-B79] Sedlazeck F. J. et al (2018) ‘Accurate Detection of Complex Structural Variations Using Single-Molecule Sequencing’, Nature Methods , 15: 461–8. 29713083 10.1038/s41592-018-0001-7 PMC5990442

[vez060-B80] Sijmons S. et al (2015) ‘High-Throughput Analysis of Human Cytomegalovirus Genome Diversity Highlights the Widespread Occurrence of Gene-Disrupting Mutations and Pervasive Recombination’, Journal of Virology, 89: 7673–95. 25972543 10.1128/JVI.00578-15 PMC4505652

[vez060-B81] Simón O. et al (2006) ‘Dynamics of Deletion Genotypes in an Experimental Insect Virus Population’, Proceedings of the Royal Society B: Biological Sciences, 273: 783–90. 10.1098/rspb.2005.3394 PMC1560231 16618670

[vez060-B82] Slack J., Arif B. M. (2006). ‘The Baculoviruses Occlusion‐Derived Virus: Virion Structure and Function’, Advances in Virus Research, 69: 99–165. 10.1016/S0065-3527(06)69003-9 PMC7112300 17222693

[vez060-B83] Sommer D. D. et al (2007) ‘Minimus: A Fast, Lightweight Genome Assembler’, BMC Bioinformatics, 8: 64. 17324286 10.1186/1471-2105-8-64 PMC1821043

[vez060-B84] Sparks W., Li H., Bonning B. (2008). ‘Protocols for Oral Infection of Lepidopteran Larvae with Baculovirus’, Journal of Visualized Experiments. 19: 888. 10.3791/888 PMC2872978 19066541

[vez060-B85] Stöcker B. K., Köster J., Rahmann S. (2016) ‘SimLoRD: Simulation of Long Read Data’, Bioinformatics, 32: 2704–6. 27166244 10.1093/bioinformatics/btw286

[vez060-B86] Szpara M. L. et al (2014) ‘Evolution and Diversity in Human Herpes Simplex Virus Genomes’, Journal of Virology, 88: 1209–27. 24227835 10.1128/JVI.01987-13 PMC3911644

[vez060-B87] Tallon L. J. et al (2014) ‘Single Molecule Sequencing and Genome Assembly of a Clinical Specimen of *Loa loa*, the Causative Agent of Loaiasis’, BMC Genomics, 15: 788. 25217238 10.1186/1471-2164-15-788 PMC4175631

[vez060-B88] Tcherepanov V., Ehlers A., Upton C. (2006) ‘Genome Annotation Transfer Utility (GATU): Rapid Annotation of Viral Genomes Using a Closely Related Reference Genome’, BMC Genomics, 7:150. 16772042 10.1186/1471-2164-7-150 PMC1534038

[vez060-B89] Thézé J. et al (2015) ‘Gene Acquisition Convergence between Entomopoxviruses and Baculoviruses’, Viruses, 7: 1960–74. 25871928 10.3390/v7041960 PMC4411684

[vez060-B90] Toussaint E. F. A. et al (2012) ‘Palaeoenvironmental Shifts Drove the Adaptive Radiation of a Noctuid Stemborer Tribe (Lepidoptera, Noctuidae, Apameini) in the Miocene’, PLoS One, 7: e41377. 22859979 10.1371/journal.pone.0041377 PMC3409182

[vez060-B91] Treangen T. J. et al (2011) ‘Next Generation Sequence Assembly with AMOS’, Current Protocols in Bioinformatics, 33: 11.8. 10.1002/0471250953.bi1108s33 PMC3072823 21400694

[vez060-B92] Tsai I. J. et al (2014) ‘Summarizing Specific Profiles in Illumina Sequencing from Whole-Genome Amplified DNA’, DNA Research, 21: 243–54. 24353264 10.1093/dnares/dst054 PMC4060946

[vez060-B93] van Oers M., Vlak J. (2007) ‘Baculovirus Genomics’, Current Drug Targets, 8: 1051–68. 17979665 10.2174/138945007782151333

[vez060-B94] Vasilijevic J. et al (2017) ‘Reduced Accumulation of Defective Viral Genomes Contributes to Severe Outcome in Influenza Virus Infected Patients’, PLoS Pathogens, 13: e1006650. 29023600 10.1371/journal.ppat.1006650 PMC5638565

[vez060-B95] Walker B. J. et al (2014) ‘Pilon: An Integrated Tool for Comprehensive Microbial Variant Detection and Genome Assembly Improvement’, PLoS One, 9: e112963. 25409509 10.1371/journal.pone.0112963 PMC4237348

[vez060-B96] Ward J. H. (1963) ‘Hierarchical Grouping to Optimize an Objective Function’, Journal of the American Statistical Association, 58: 236–44.

[vez060-B97] Wei Z.-G., Zhang S.-W. (2018) ‘NPBSS: A New PacBio Sequencing Simulator for Generating the Continuous Long Reads with an Empirical Model’, BMC Bioinformatics, 19:177. 29788930 10.1186/s12859-018-2208-0 PMC5964698

[vez060-B98] Wong K. et al (2010) ‘Enhanced Structural Variant and Breakpoint Detection Using SVMerge by Integration of Multiple Detection Methods and Local Assembly’, Genome Biology, 11: R128. 21194472 10.1186/gb-2010-11-12-r128 PMC3046488

[vez060-B99] Xiaofei E., Kowalik T. (2014) ‘The DNA Damage Response Induced by Infection with Human Cytomegalovirus and Other Viruses’, Viruses, 6: 2155–85. 24859341 10.3390/v6052155 PMC4036536

[vez060-B100] Ye K. et al (2009) ‘Pindel: A Pattern Growth Approach to Detect Break Points of Large Deletions and Medium Sized Insertions from Paired-End Short Reads’, Bioinformatics, 25: 2865–71. 19561018 10.1093/bioinformatics/btp394 PMC2781750

[vez060-B101] Zarate S. et al (2018). ‘Parliament2: Fast Structural Variant Calling Using Optimized Combinations of Callers’, BioRxiv.

[vez060-B102] Zhang W., Jia B., Wei C. (2019) ‘PaSS: A Sequencing Simulator for PacBio Sequencing’, BMC Bioinformatics, 20:352. 31226925 10.1186/s12859-019-2901-7 PMC6588853

[vez060-B103] Zhang Y. et al (2018) ‘Large-Scale Comparative Epigenomics Reveals Hierarchical Regulation of non-CG Methylation in *Arabidopsis*’, Proceedings of the National Academy of Sciences of the United States of America, 115: E1069–E1074. 29339507 10.1073/pnas.1716300115 PMC5798360

[vez060-B104] Zwart M. P., Tromas N., Elena S. F. (2013) ‘Model-Selection-Based Approach for Calculating Cellular Multiplicity of Infection during Virus Colonization of Multi-cellular Hosts’, PLoS One, 8: e64657. 23724074 10.1371/journal.pone.0064657 PMC3665715

