# Supplementary material for: Wide spectrum and high frequency of genomic structural variation, including transposable elements, in large double-stranded DNA viruses
Source: Virus Evol. 2020 Jan 27;6(1):vez060. doi: 10.1093/ve/vez060 (PMC6983493; doi:10.1093/ve/vez060)
Supplement: vez060_Supplementary_Data [file vez060_supplementary_data.zip › vez060-Suppl_data/Supplementary_Table_S3.docx]

Table S3: Numbers and frequencies of AcMNPV SVs detected in real and simulated short-reads. The frequencies were computed considering the number of SVs per viral genome follows a Poisson distribution.

|  | **Real data** | **Simulated data** |
| --- | --- | --- |
|  | **AcMNPV** | **AcMNPV** |
| **SV number** | 1263 | 802 |
| **Total frequency (%)** | 20.61 | 1.47 |
| **Deletion number** | 308 | 737 |
| **Duplication number** | 149 | 65 |
| **Insertion number** | 0 | 0 |
| **Inversion number** | 806 | 0 |
