# Supplementary material for: Wide spectrum and high frequency of genomic structural variation, including transposable elements, in large double-stranded DNA viruses
Source: Virus Evol. 2020 Jan 27;6(1):vez060. doi: 10.1093/ve/vez060 (PMC6983493; doi:10.1093/ve/vez060)
Supplement: vez060_Supplementary_Data [file vez060_supplementary_data.zip › vez060-Suppl_data/Suppplementary_Table_S2.docx]

Table S2: Viral regions affected by the five most frequent SVs in the AcMNPV, HCMV, IIV6 and IIV31 populations. As expected, the majority of most frequent SV breakpoints involve intergenic regions, non-essential or uncharacterized genes, these regions encoding no essential proteins for the viral life cycles. The two genes encompassing most frequent SV breakpoints in IIV6 have not known functions, in part because this virus is less studied than the two others. The frequencies are computed considering the SV number per viral genome follows a Poisson distribution.

| SVs | regions | Frequency (%) | Location of the SV start coordinate | Location of the SV end coordinate |
| --- | --- | --- | --- | --- |
| **AcMNPV** | **/** | **39.9** | **/** | |
| Insertion | hr4b | 6.03 | Homologous repeated region | / |
| Deletion | NA-NA | 3.65 | intergenic region | intergenic region |
| Deletion | NA-PNK\|PNL | 1.65 | intergenic region | Uncharacterized |
| Insertion | AcOrf-145 | 0.95 | Chitin binding | / |
| Duplication | AcOrf-145-AcOrf-145 | 0.94 | Chitin binding | Chitin binding |
| **HCMV** | **/** | **54.4** | **/** | |
| Inversion | NA-NA | 5.62 | intergenic region | intergenic region |
| Deletion | RL1-RL1 | 3.26 | Non-essential gene for viral growth | Non-essential gene for viral growth |
| Deletion | NA-NA | 3.11 | intergenic region | intergenic region |
| Deletion | NA-NA | 2.56 | intergenic region | intergenic region |
| Duplication | NA-NA | 2.51 | intergenic region | intergenic region |
| **IIV6** | **/** | **52.4** | **/** | |
| Deletion | 444-444 | 13.9 | Uncharacterized | Uncharacterized |
| Deletion | 444-444 | 12.4 | Uncharacterized | Uncharacterized |
| Deletion | 444-444 | 11.9 | Uncharacterized | Uncharacterized |
| Deletion | 444-444 | 3.9 | Uncharacterized | Uncharacterized |
| Deletion | 444-444 | 3.1 | Uncharacterized | Uncharacterized |
| **IIV31** | **/** | **80.1** | **/** | |
| Deletion | 34R-NA | 37.3 | Hypothetical protein | intergenic region |
| Deletion | NA-122R | 32.8 | intergenic region | Hypothetical protein |
| Deletion | 120R-120R | 24.8 | DNA-directed RNA polymerase subunit | DNA-directed RNA polymerase subunit |
| Deletion | NA-077R | 23.8 | intergenic region | Bro-like protein, GIY-YIG domain |
| Insertion | NA-NA | 6.6 | intergenic region | intergenic region |
