# Supplementary material for: Wide spectrum and high frequency of genomic structural variation, including transposable elements, in large double-stranded DNA viruses
Source: Virus Evol. 2020 Jan 27;6(1):vez060. doi: 10.1093/ve/vez060 (PMC6983493; doi:10.1093/ve/vez060)
Supplement: vez060_Supplementary_Data [file vez060_supplementary_data.zip › vez060-Suppl_data/Supplementary_Figures.docx]

**Supplementary figures**


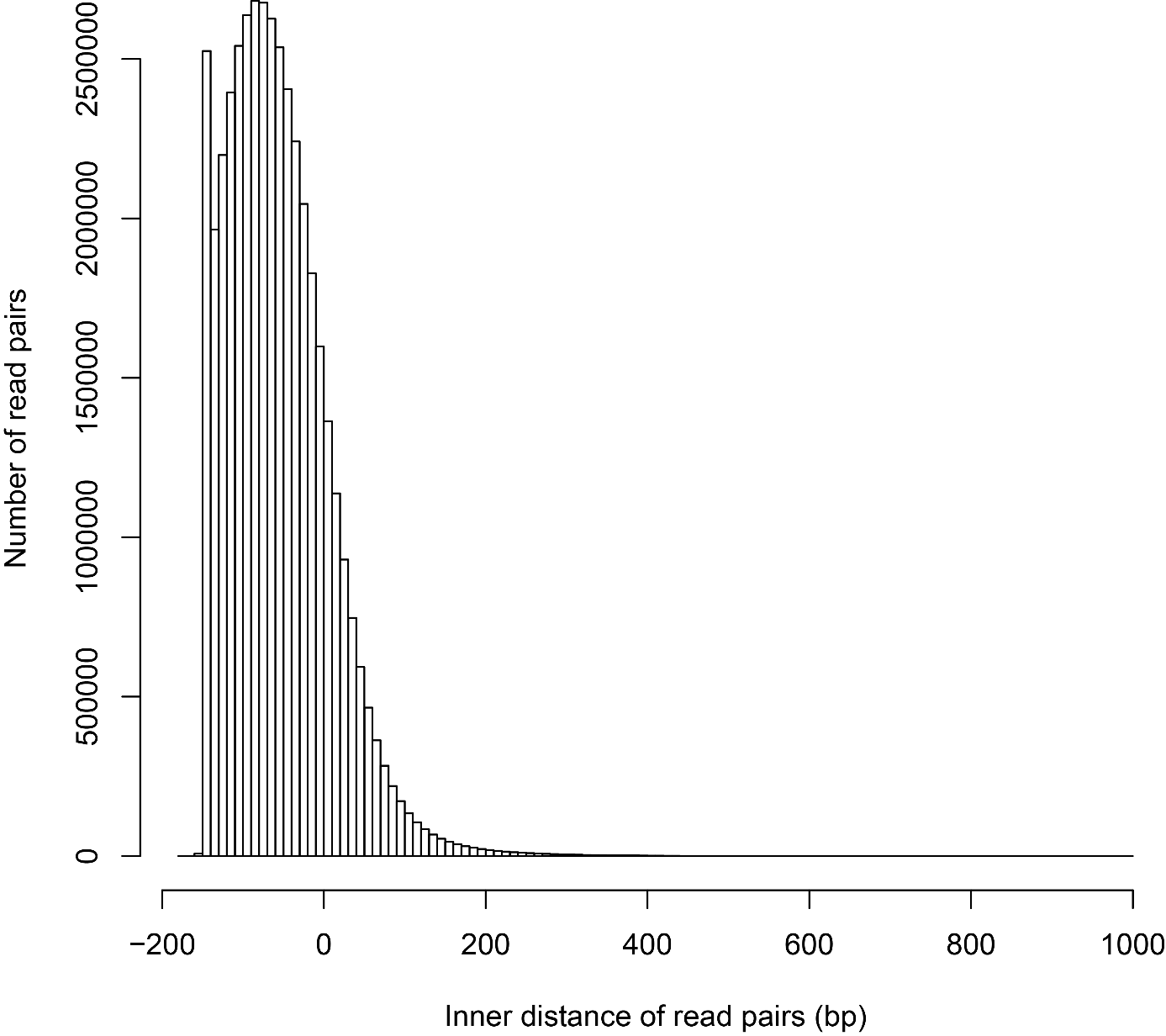


Figure S1: Distribution of inner distance of all Illumina read pairs aligning on the AcMNPV genome. Most of the pairs have an inner distance <700bp, the threshold used to consider the distance as the result of a large deletion. The inner distance can be negative when both reads of a pair overlap.


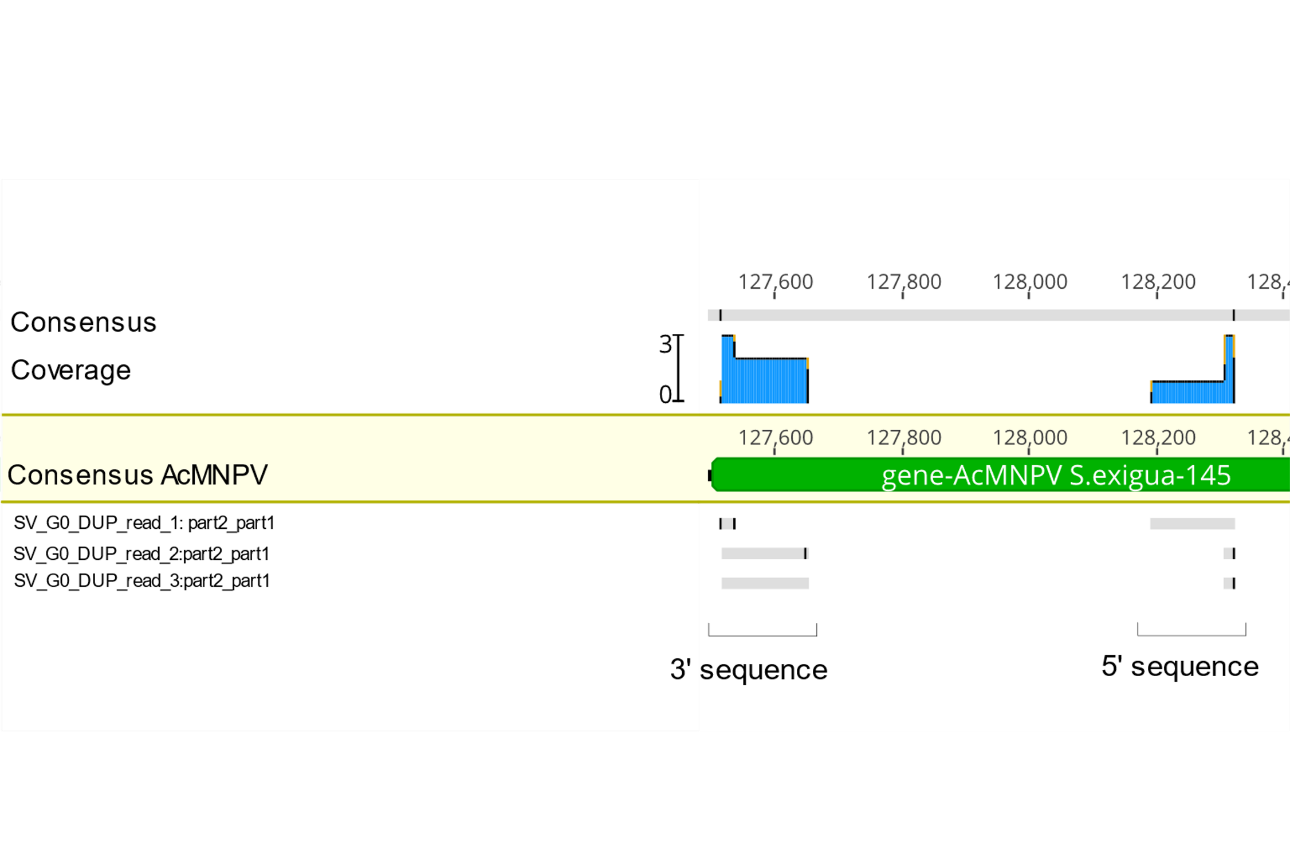


Figure S2: Duplication detected in three reads in the AcMNPV G0 data. The reads are split in a way supporting a duplication event. This duplication was detected in only three reads, supporting SV detection by three reads as true events and emphasizing the precision of our frequency calculation.


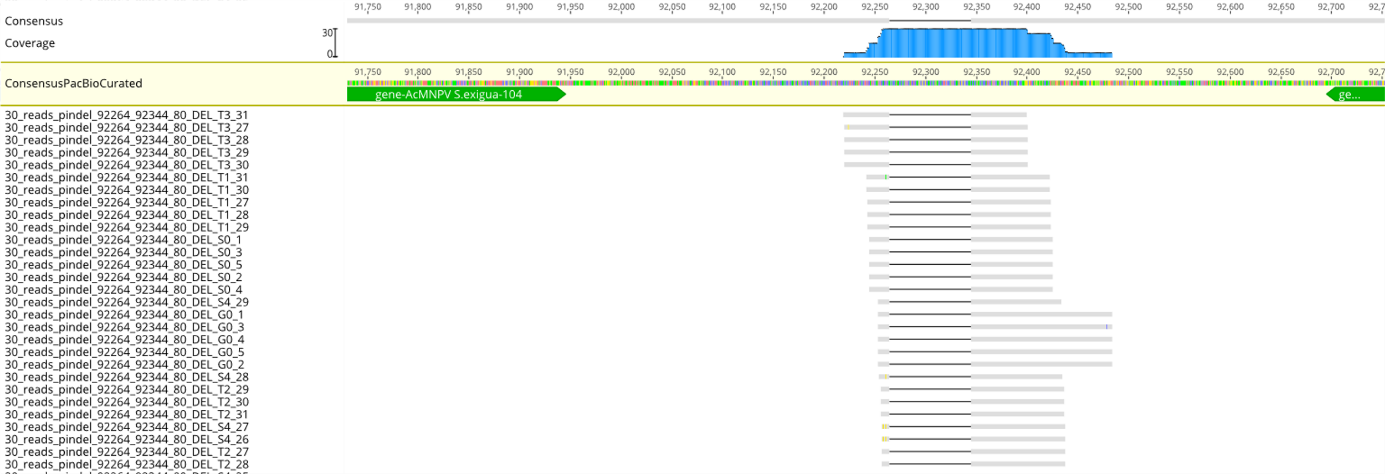


Figure S3: The most frequent 'NA_NA' deletion detected in short reads from the AcMNPV data. The short deletion is visible into each read with a precise alignment. Only a sample of all reads supporting the deletion is visible here.


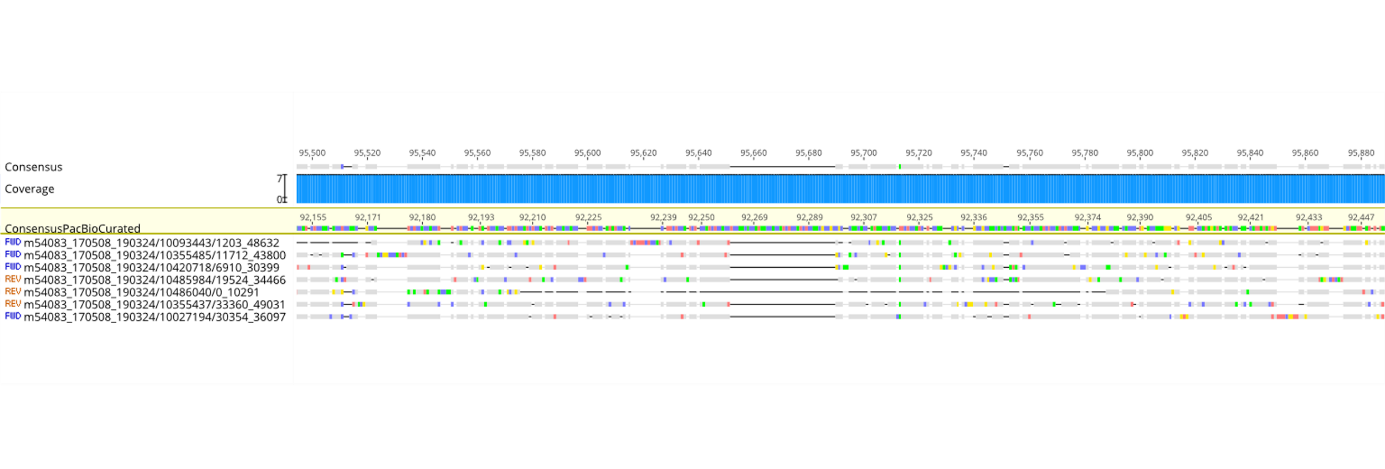


Figure S4: The most frequent 'NA_NA' deletion detected in long reads from the AcMNPV data. Only some reads supporting the deletion were retrieved here. As expected, the high error rate of long-read sequencing hinders the detection of SV breakpoint at a nucleotide resolution. Regarding the fifth read with a larger deletion in the alignment, our clustering step can gather reads with a deletion of different sizes, likely two different deletion events. That is why our approach is conservative in the number of detected SVs, likely greater than the ones we detected.


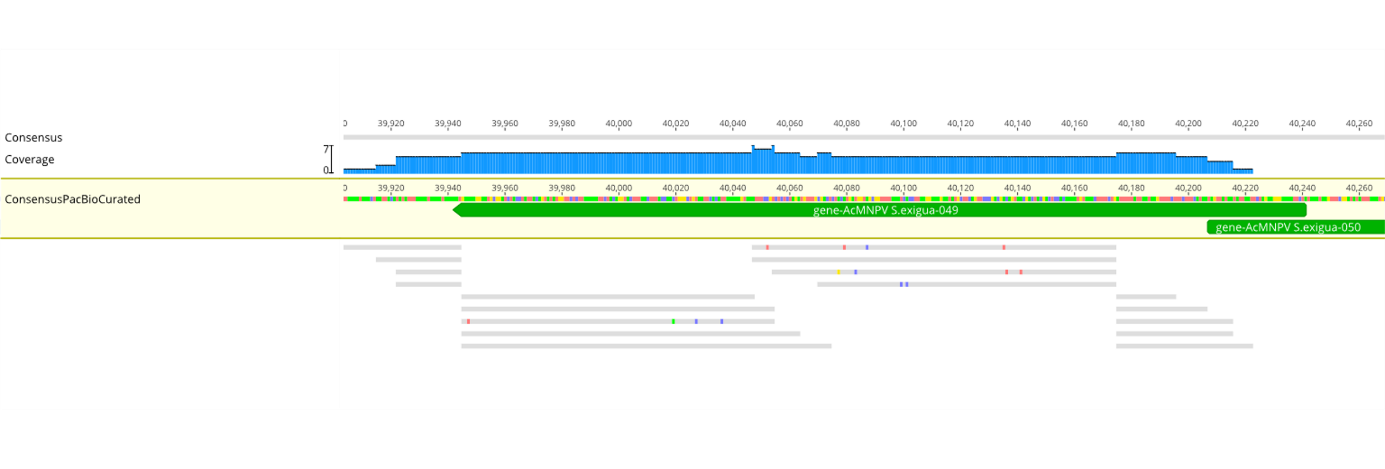


Figure S5: Inversion detected in short reads from the AcMNPV data. Short reads can also detect inversion with a high precision. This inversion is supported by nine reads and occurred into a viral gene.


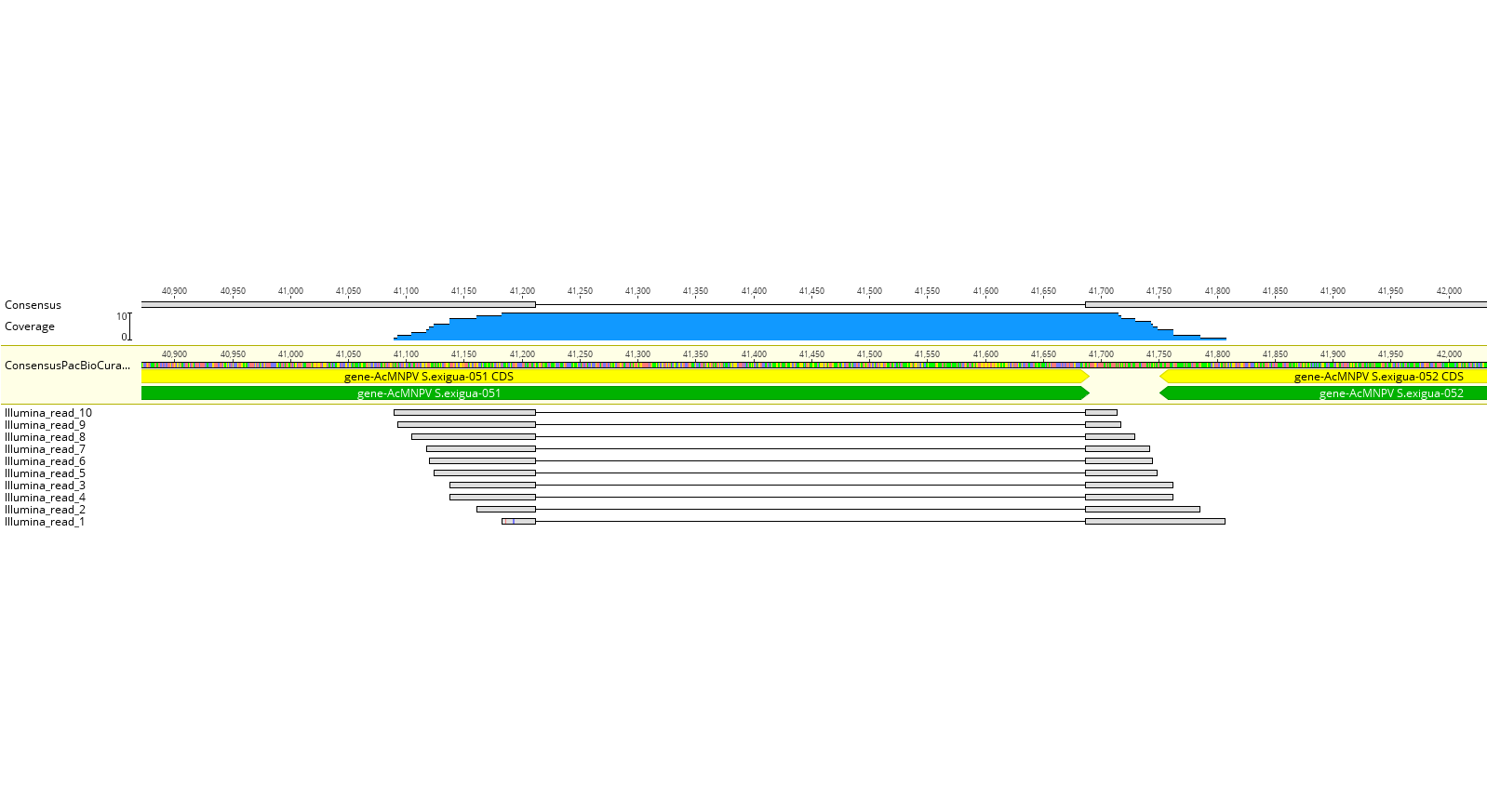


Figure S6: Deletion detected in short reads from the AcMNPV data. This deletion is supported by ten reads and occurred into a viral gene.


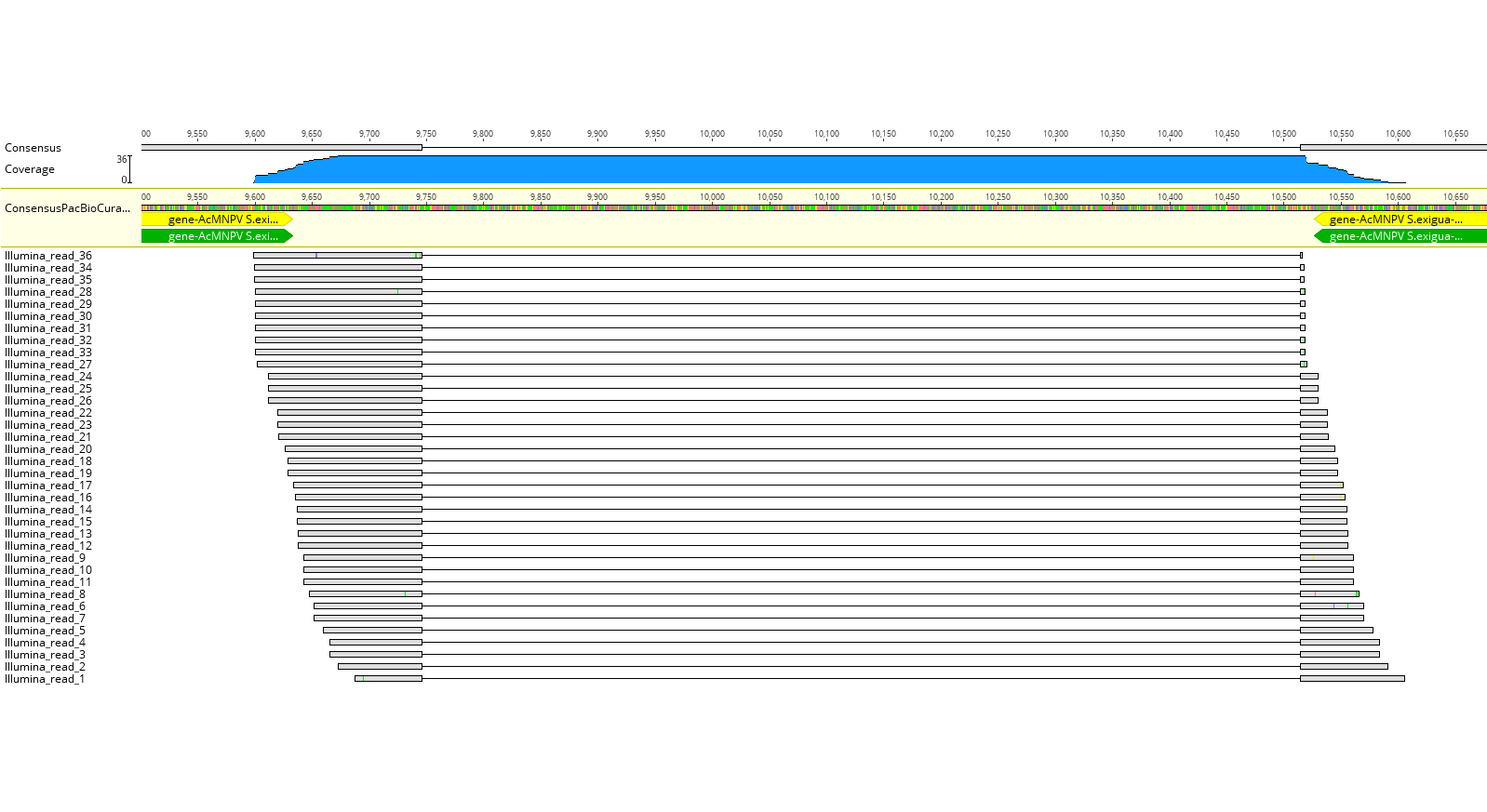


Figure S7: Deletion detected in short reads from the AcMNPV data. This deletion is supported by 36 reads and occurred into an intergenic region.


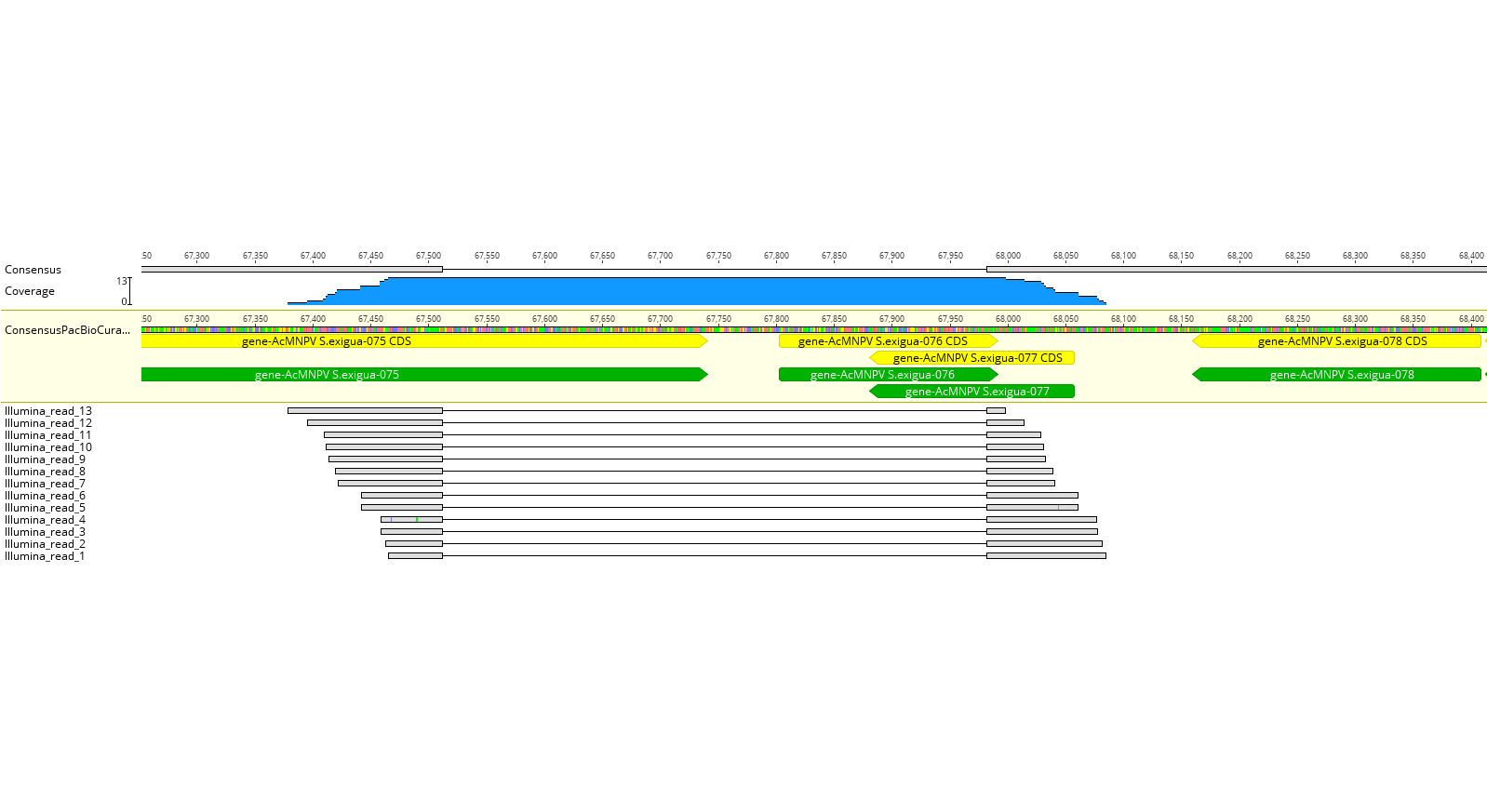


Figure S8: Deletion detected in short reads from the AcMNPV data. This deletion is supported by 13 reads and involved two genes.


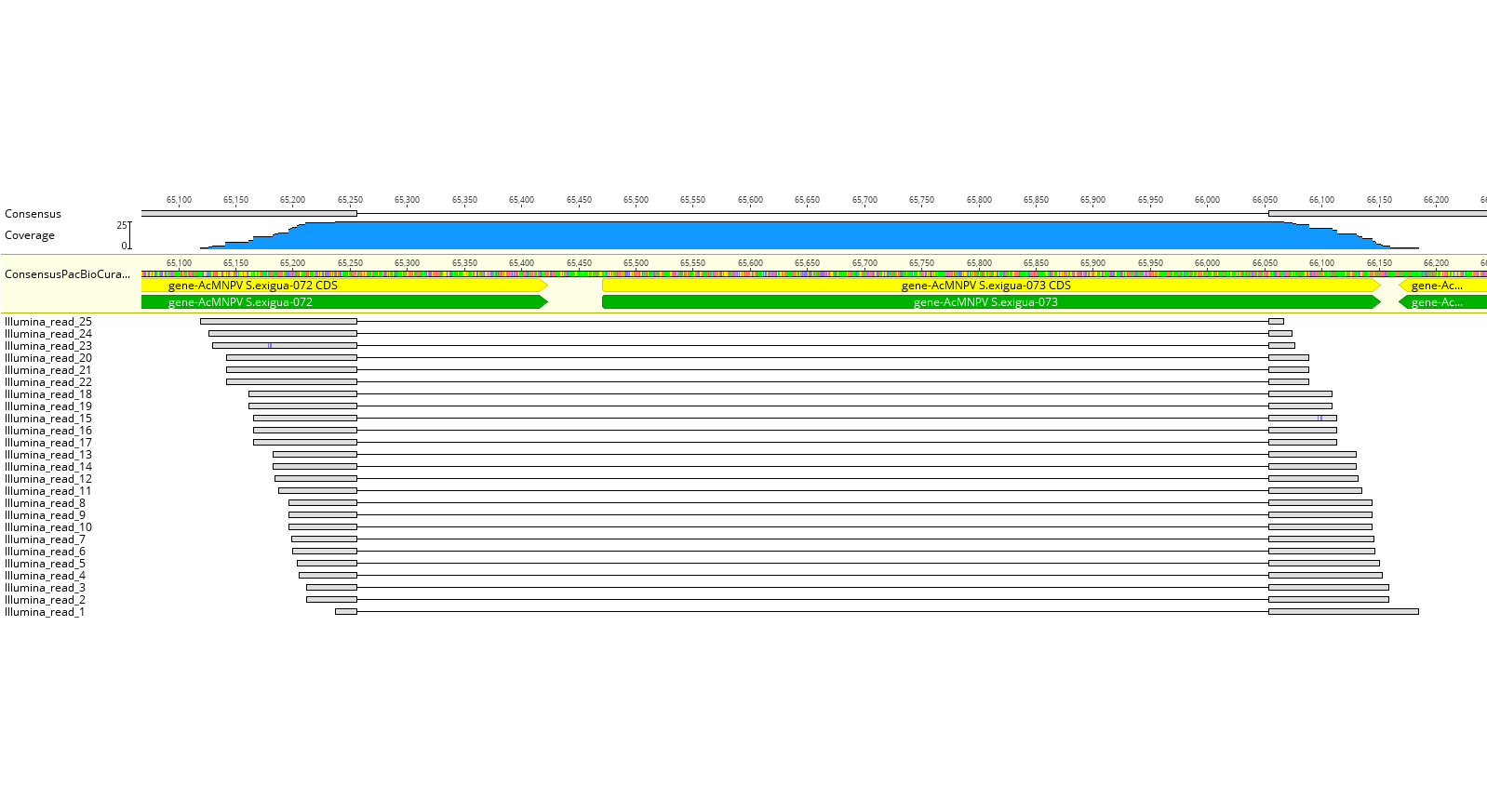


Figure S9: Deletion detected in short reads from the AcMNPV data. This deletion is supported by 25 reads and involved two genes.


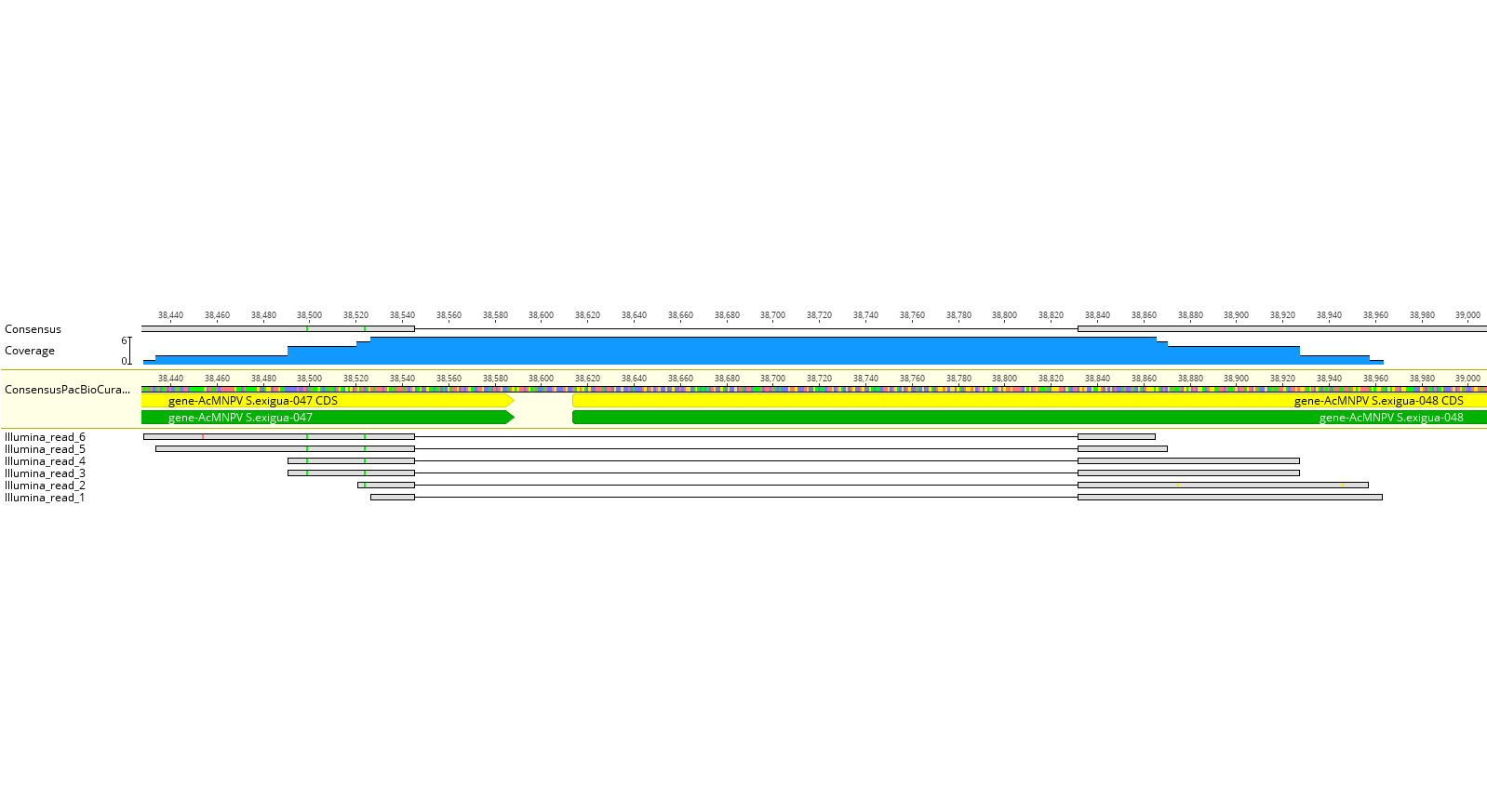


Figure S10: Deletion detected in short reads from the AcMNPV data. This deletion is supported by six reads and involved two genes.


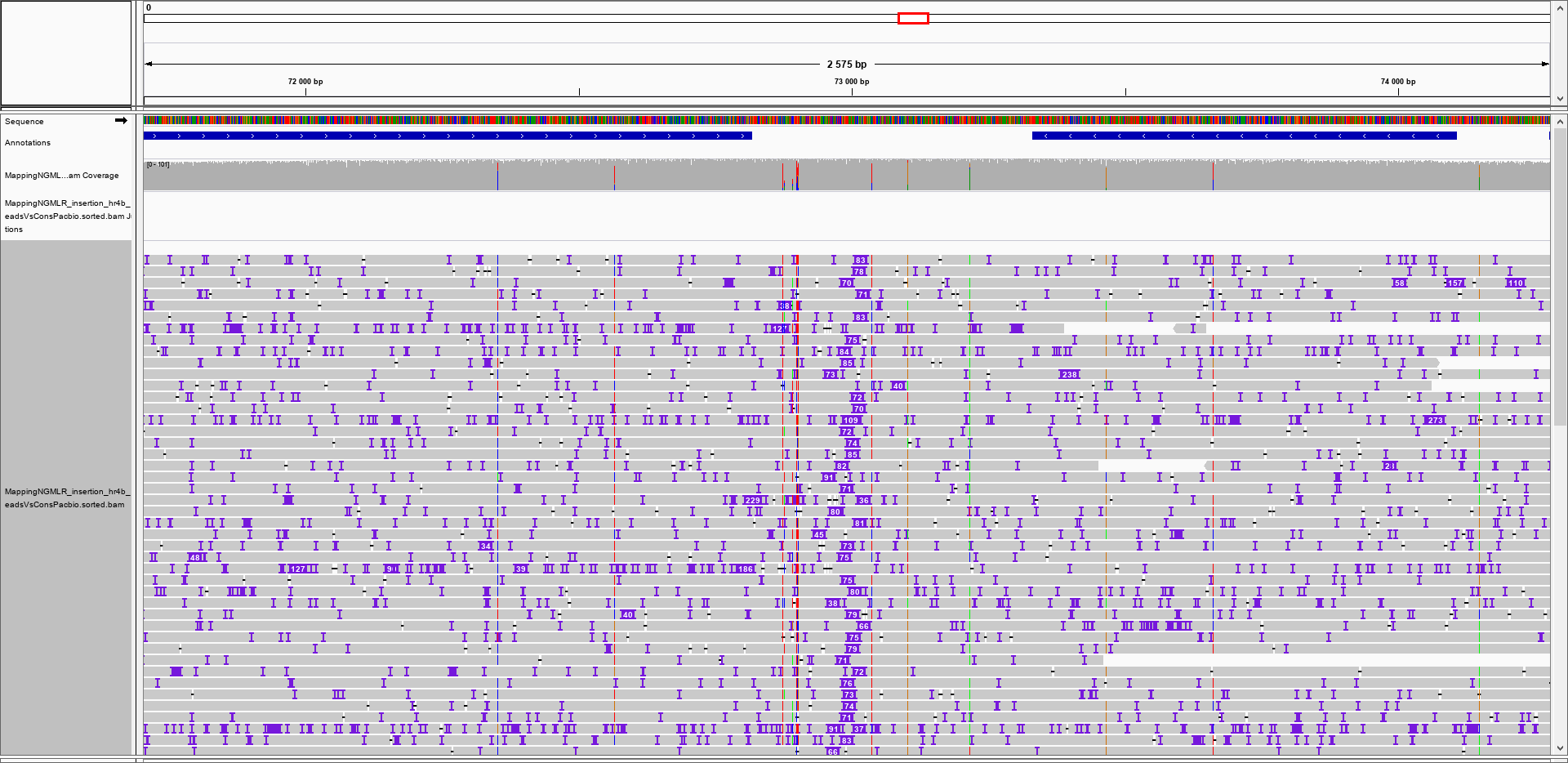


Figure S11: Insertion detected in long reads from the AcMNPV data. Long reads can also detect insertion. This insertion is supported by many reads and occurred into the hr4b region. The read alignment is visualized with IGV. Insertions are represented as purple rectangles indicating the number of inserted base pairs. The inserted length corresponds to about 70 bp.


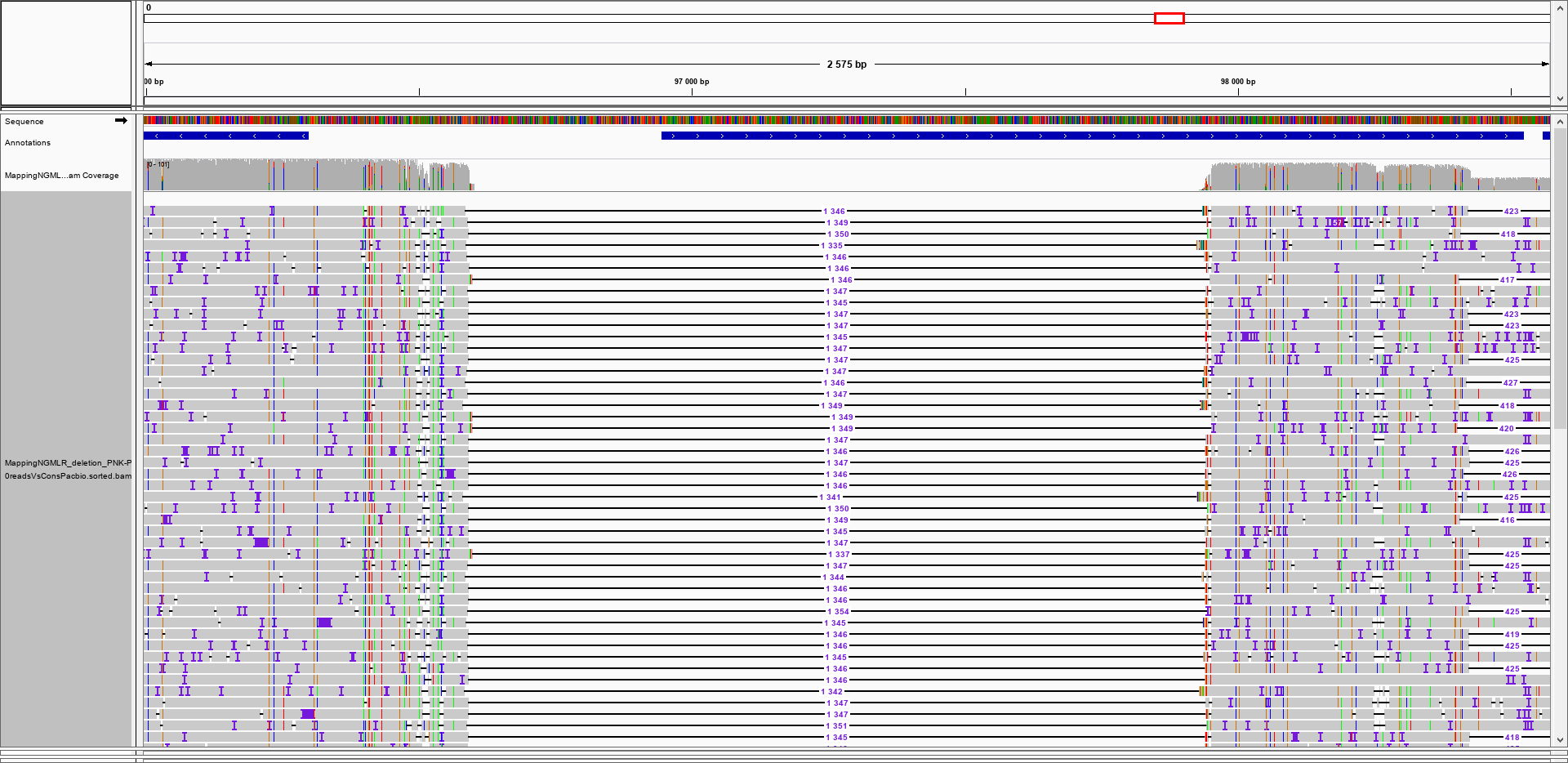


Figure S12: Deletion detected in long reads from the AcMNPV data. This deletion occurs between an intergenic region and the PNK|PNL gene. The read alignment is visualized with IGV. The numbers indicate the deletion length, about 1,347 bp.


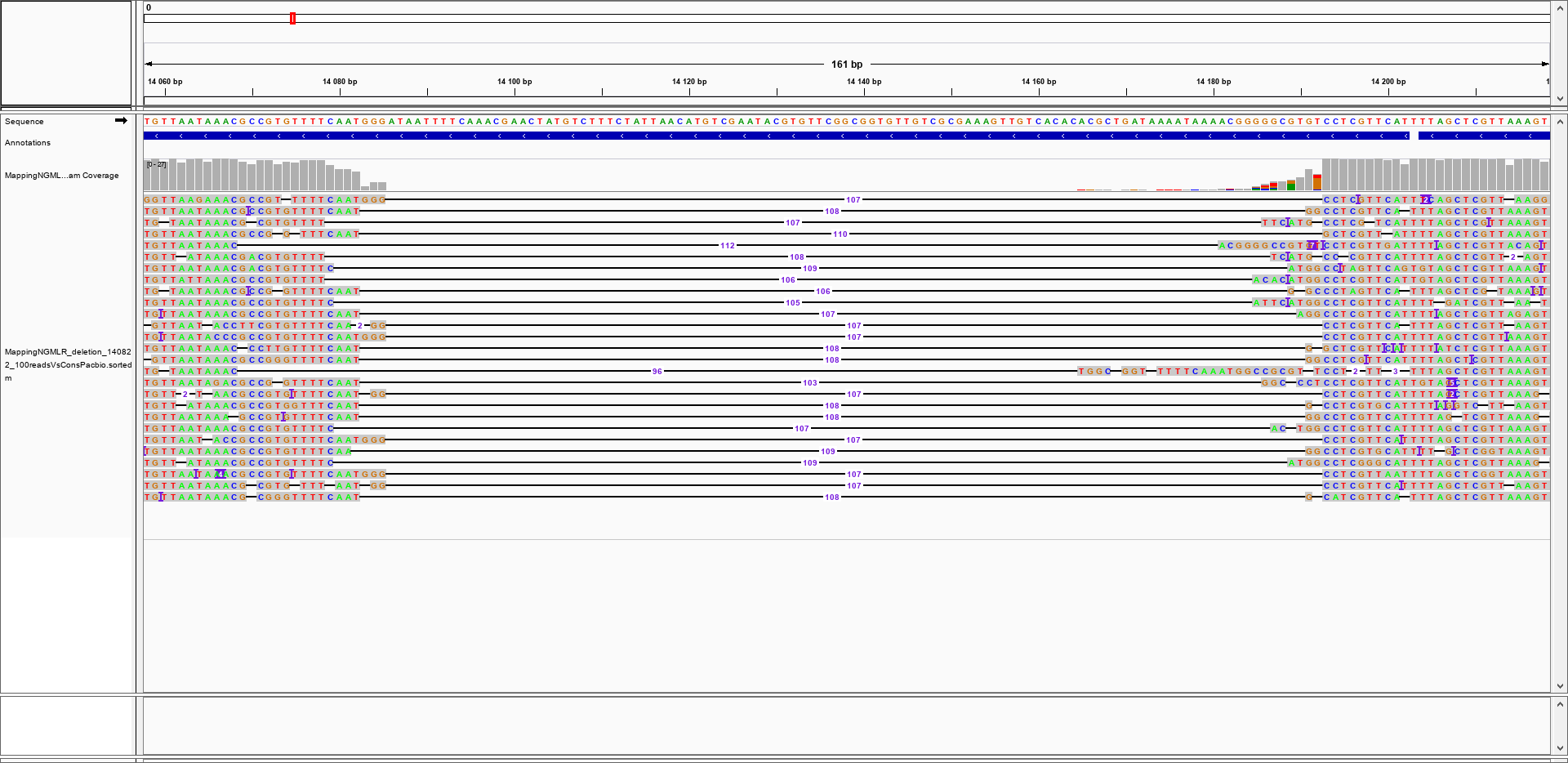


**Figure S13: Deletion detected in long reads from the AcMNPV data.** This deletion occurs in the Ac-IAP1 gene. The read alignment is visualized with IGV. The numbers indicate the deletion length, about 108 bp.


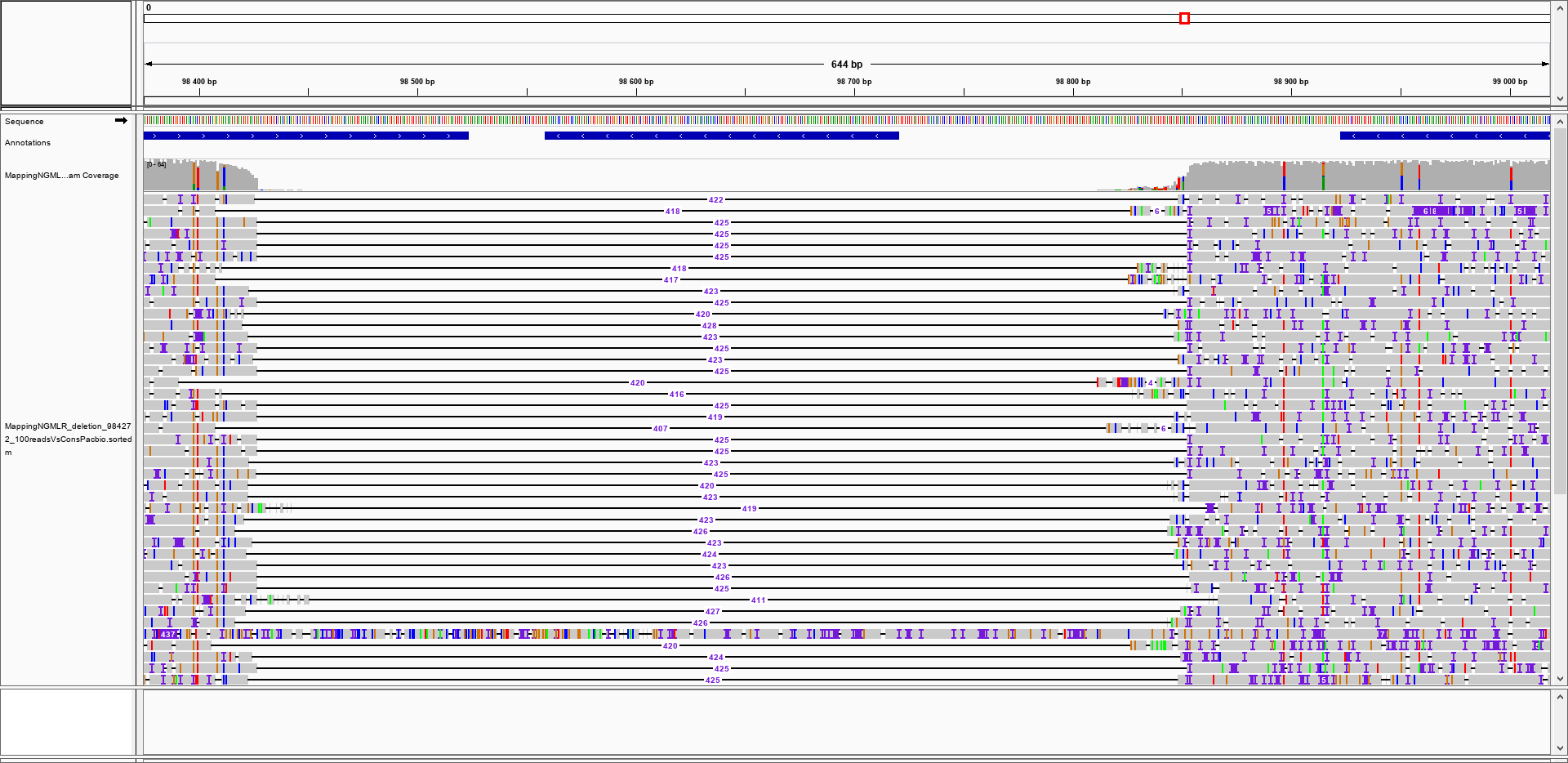


**Figure S14: Deletion detected in long reads from the AcMNPV data.** This deletion begins into the Ac-PNK|PNL gene and encompasses the AcOrf-85 gene.The read alignment is visualized with IGV. The numbers indicate the deletion length, about 425 bp.


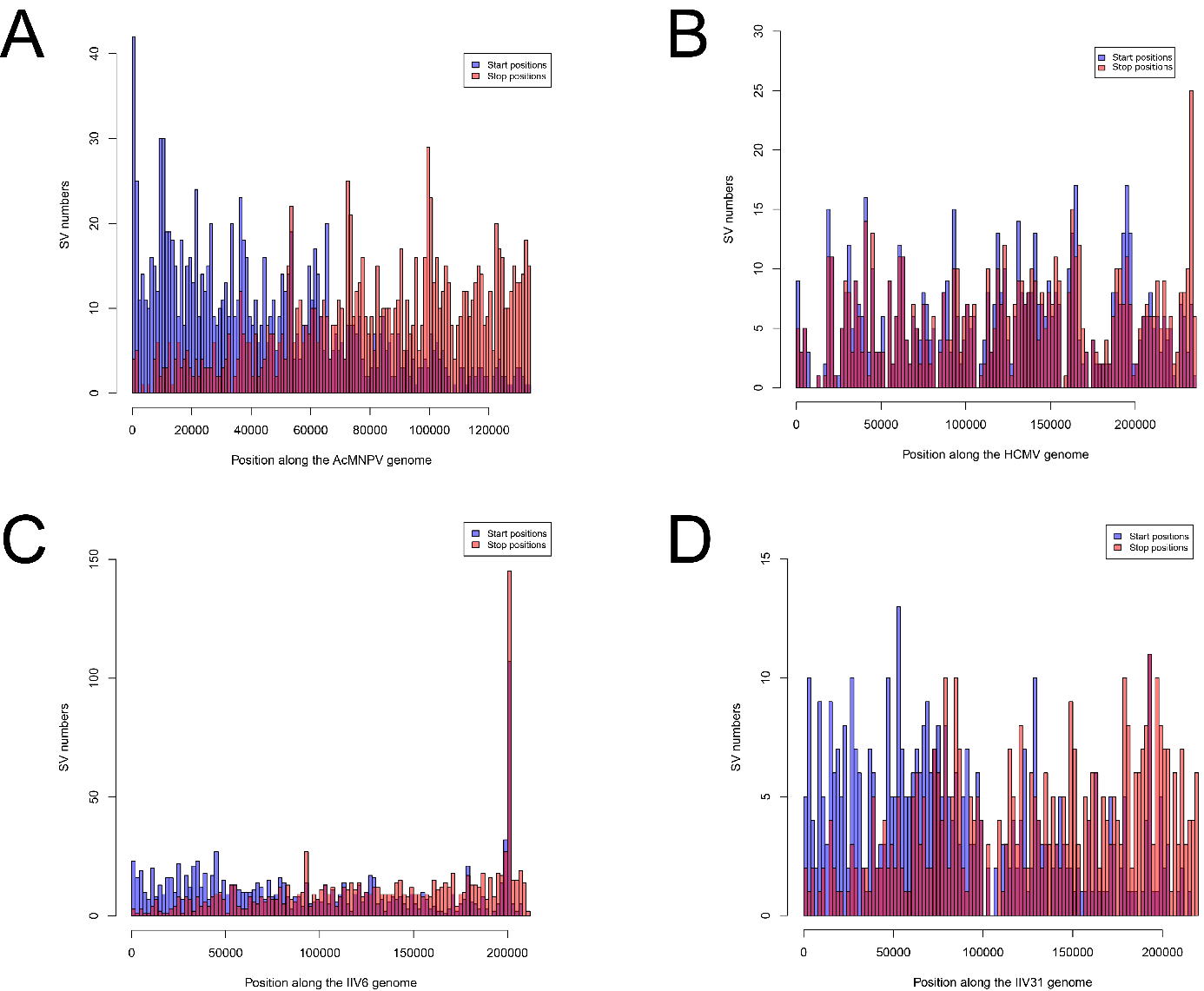


Figure S15: SV breakpoint positions along the viral genomes. Breakpoints are found all along the viral genomes. A: Breakpoint positions of detected SVs along the AcMNPV genome. B: Breakpoint positions of detected SVs along the HCMV genome. C: Breakpoint positions of detected SVs along the IIV6 genome. D: Breakpoint positions of detected SVs along the IIV31 genome.


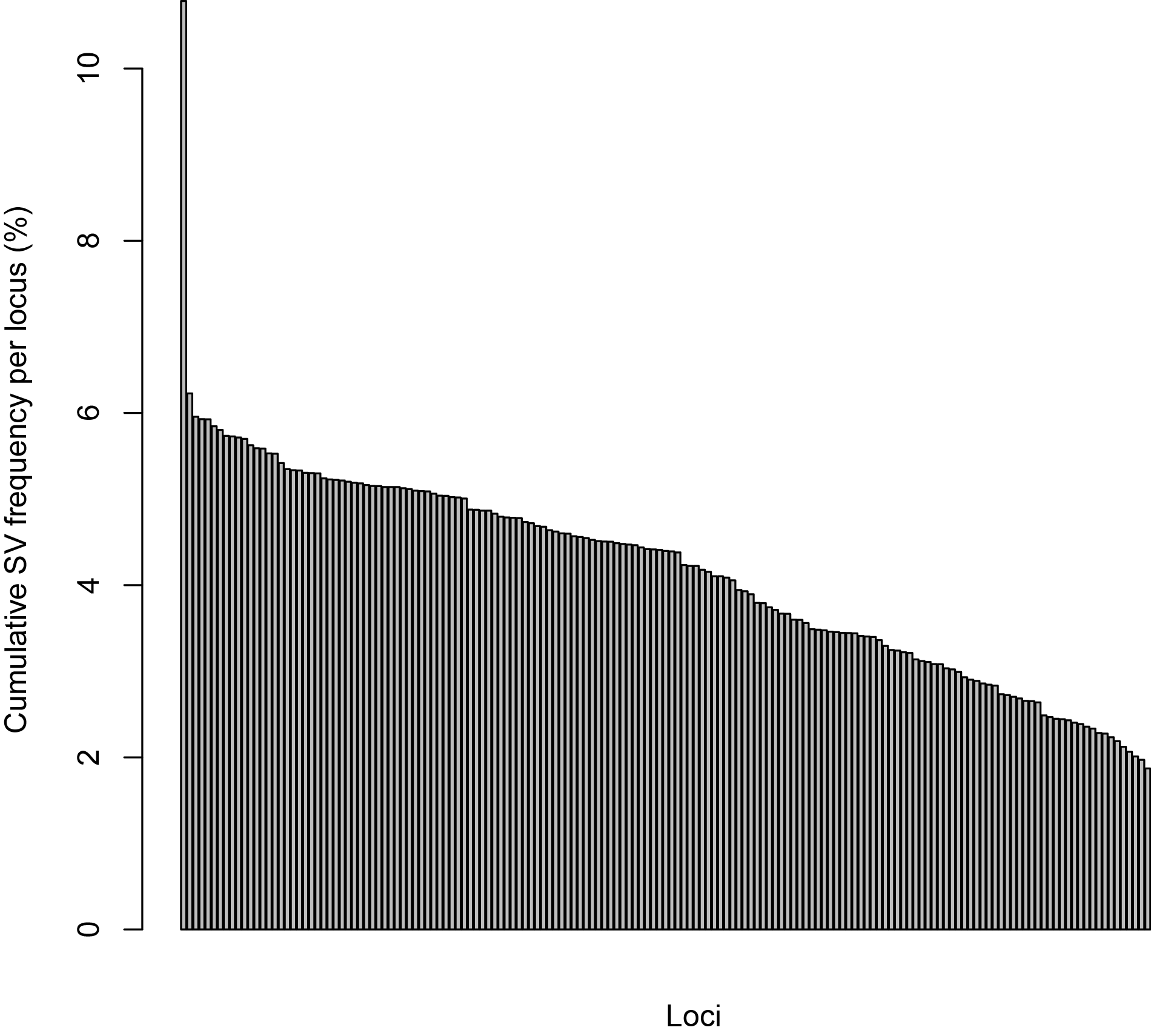


Figure S16: Cumulative frequency of SVs affecting each locus of the AcMNPV genome. The frequency was computed assuming the number of SVs per viral genome follow a Poisson distribution. Most of loci have an SV frequency ranging from 1.9% to 6.2%.


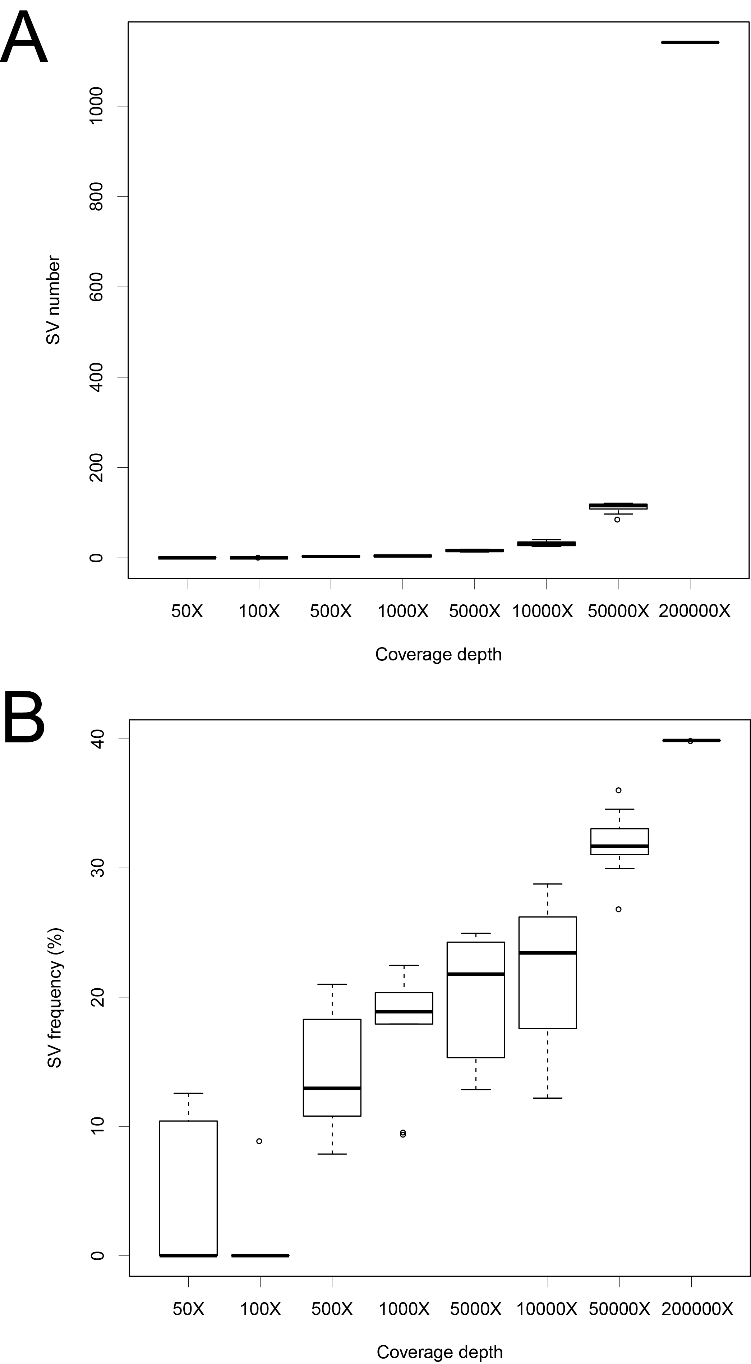


**Figure S17: Number and frequency of detected SVs according to the coverage depth.** **A:** SV number detected for each coverage depth. **B:** Cumulative mean frequency computed for each coverage depth. Frequencies were computed assuming the number of SVs per viral genome follows a Poisson distribution. On the two graphs, the SV number and frequency computed with the full data (200,000X) were added for comparison. The subsampling of AcMNPV long-read and short-read datasets was performed to get a number of reads corresponding to 50X, 100X, 500X, 1,000X, 5,000X, 10,000X and 50,000X coverage on the AcMNPV genome with ten replicates for each coverage depth. The SV detection was performed just as for the entire data (200,000X). Paired Wilcoxon tests showed SV numbers were always statistically different (excepted between 50X and 100X) between 100X and 500X; 500X and 1,000X; 1,000X and 5,000X; 5,000X and 10,000X and 10,000X and 50,000X (Wilcoxon tests, W=63, p-value=0.2113; W=1.5, p-value=0.0001218; W=23, p-value=0.03849; W=0, p-value=0.0001697; W=0, p-value=0.0001786; W=100, p-value=0.00001083; Kruskall-Wallis X²=66.207, df=6, p-value=2.444*10^-12^). Wilcoxon tests with SV frequency gave the same general pattern , excepted SV frequency between 50X and 100X; 500X and 1,000X, 1,000X and 5,000X and 5,000X and 10,000X were not significantly different (Wilcoxon tests, W=67, p-value=0.1012; W=1, p-value=0.0001212; W=33, p-value=0.2176; W=34, p-value=0.2475; W=34, p-value=0.2475; W=2, p-value=0.0000433; Kruskall-Wallis X²=55.53, df=6, p-value=3.622*10^-10^). These results suggest the global number and frequency of SVs depend on coverage depth, due to a high number of SVs supported by only few reads, concordant with the very low SV frequency in the viral population. A coverage depth below 5,000X does not allow an accurate detection in terms of number and frequency of SVs, due to a too small set of viral genomic data. We confirmed there was a high homogeneity in SV number and frequency among replicates (SV number: Kruskall-Wallis X²=0.39001, df=9, p-value=1; SV frequency: Kruskall-Wallis X²=1.675, df=9, p-value=0.9956). It is worthy to note the detection of an insertion of ~70bp located in the hr2 region that was present in ~10% of viral genomes, depending the dataset. This was the most frequent SV detected in most of the datasets and in all the 10,000X and 50,000X datasets. This insertion was detected at all the coverage depth tested here. Strangely, it was not detected in our SV dataset with all the reads. To be sure the problem did not come from our clustering step, we searched for the presence of this insertion in the VCF output files from the SV callers that detected it, Pindel and Sniffles programs. We found the insertion was present in the Pindel output from the subsampled data and from the entire data (200,000X). However, the insertion was present in the Sniffles output from the subsampled data but not from the entire data. The large amount of data with all long or short reads could hamper the optimal use of SV callers and lead in some case to a lack of detection of some SVs.
